# MOF-303 with Lowered Water Evaporation Enthalpy for Solar Steam Generation

**DOI:** 10.1021/acsami.4c10506

**Published:** 2024-09-06

**Authors:** Yi-Hsuan Lin, Hsun-Hao Lin, Yu-Shuo Lee, Wen-Yueh Yu, Shyh-Chyang Luo, Dun-Yen Kang

**Affiliations:** † Department of Chemical Engineering, National Taiwan University, No. 1, Sec. 4, Roosevelt Road, Taipei 106319, Taiwan; ‡ Department of Materials Science and Engineering, National Taiwan University, No. 1, Sec. 4, Roosevelt Road, Taipei 106319, Taiwan

**Keywords:** metal−organic framework, MOF-303, Janus membrane, solar steam generation, desalination

## Abstract

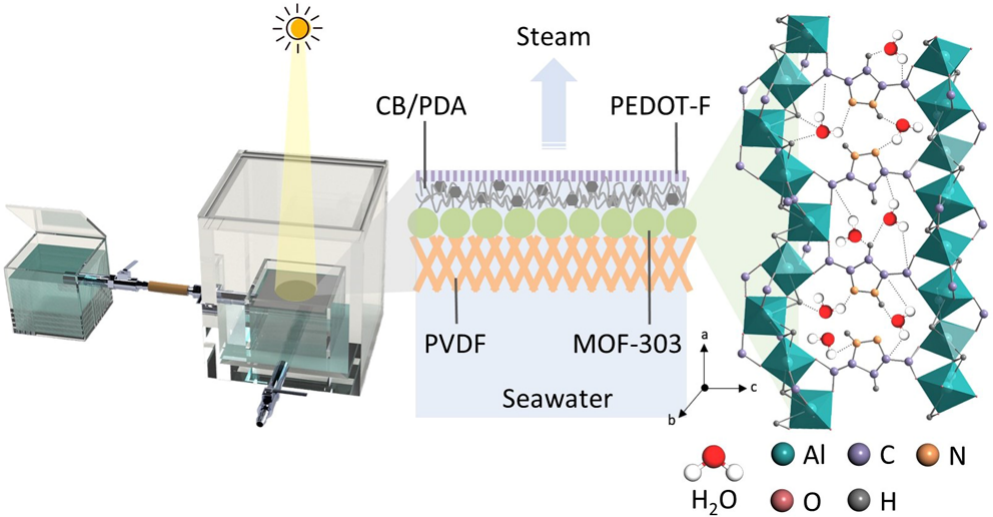

Hydrophilic metal–organic frameworks (MOFs) are promising for solar steam generation from waste or seawater. In this study, we propose a MOF-based Janus membrane for efficient solar steam generation. We selected MOF-303 for its hydrophilic properties and 1D channels with 6.5 Å cavity diameter, making it an excellent water-absorbing layer. Characterization via Raman spectroscopy and differential scanning calorimetry indicates that the nanoconfinement within MOF-303 can reduce the water evaporation enthalpy, thereby boosting water production efficiency. When deposited on various substrates, MOF-303 aimed to optimize solar steam generation. We enhanced the membrane performance by incorporating carbon black (CB), polydopamine (PDA), and perfluoro-functionalized poly(3,4-ethylenedioxythiophene) (PEDOT-F), materials known for their solar-to-thermal energy conversion capabilities. PEDOT-F, in particular, also served as a hydrophobic layer, preventing salt recrystallization during seawater operation. Under one sun irradiation, the water evaporation flux for deionized water increased from 0.31 to 0.79 kg h^–1^ m^–2^ using a porous hydrophilic poly(vinylidene difluoride) substrate and further to 2.36 kg h^–1^ m^–2^ with the optimized MOF-303-CB/PDA-PEDOT-F membrane, achieving an energy conversion efficiency of 97%. Additionally, the desalination capability of the MOF-303 membrane effectively reduced metal ion concentrations (Na^+^, K^+^, Mg^2+^, and Ca^2+^) to meet the WHO drinking water standards. These findings demonstrate the significant potential of the MOF-303-based Janus membrane for practical applications in solar steam generation and desalination, combining high water evaporation rates with excellent energy conversion efficiency.

## 1 Introduction

Metal–organic frameworks (MOFs) are a type of crystalline material made up of metal ions or clusters bound to organic ligands, resulting in a porous three-dimensional structure. The combination of these metal ions and organic ligands offers a wide range of potential structures and compositions for MOF compounds. A key characteristic of MOFs is their high surface area and porosity, which stems from the spaces and channels between the metal–organic building blocks, forming an intricate network of interconnected pores at the nanometer or even subnanometer scale. These well-defined nanopores make MOFs promising for various applications such as gas storage,^1−5^ sensing,^6−10^ catalysis,^11−14^ and drug delivery.^15−18^ Furthermore, MOFs can be transformed into thin films or membranes, broadening their utility to include gas separation, pervaporation, and desalination. Dense layers of MOFs can be grown on porous ceramic substrates through seeded growth to enhance mass transport in different applications. These dense MOF membranes have been successfully employed in gas separations.^19−24^ Hydrophilic and water-stable MOF membranes have also demonstrated effectiveness in pervaporation, facilitating the separation of water from various alcohols.^25−27^

As the water scarcity has drawn an increasing attention in recent years, MOFs with high water adsorption uptakes have been used in the device for interfacial solar steam generation. Hu et al.^28^ pioneered the utilization of CAU-10-H as a water sorbent in conjunction with Ti_2_O_3_@ hydroxymethyl functionalized poly(3,4-ethylenedioxythiophene) as a solar absorber, integrated onto a hydrophilic polytetrafluoroethylene substrate, to create a highly efficient MOF-based solar steam generator. Their device achieved an impressive evaporation rate of 2.2 kg m^–2^ h^–1^ and an efficiency of 98%, even after a month, demonstrating its robustness. He et al.^29^ employed Co-CAT MOF and poly(vinyl alcohol) on cotton cloth to fabricate an evaporator capable of simultaneous wastewater purification and freshwater production. Leveraging Co-CAT’s photothermal conversion prowess, their evaporator exhibited a high water evaporation rate of 2.2 kg m^–2^ h^–1^, exceptional sunlight absorption nearing 97%, and efficient degradation of contaminants such as tetracycline (91.1%) under one sun irradiation. Jiang et al.^30^ introduced a novel strategy involving the chemical transformation of Bi-MOF into a Bi-C nanostructure integrated onto a carbon felt substrate. This innovative Bi-C/CF composite demonstrated superior light-harvesting capabilities and rapid water transmission, facilitating efficient solar water evaporation. Impressively, under one sun irradiation, it exhibited a high evaporation rate of 1.50 kg m^–2^ h^–1^ and an efficiency of 91.9%, alongside exceptional long-term durability. Meng et al.^31^ devised a double-boost solar energy-driven evaporator by brush-printing carbonized ZIF-8 onto a highly hydrated cellulose network wood sponge. ZIF-8′s high porosity and solar absorbent-precursor properties facilitated rapid vapor generation, resulting in an average water evaporation rate of 1.42 kg m^–2^ h^–1^. Their device achieved a daily drinkable freshwater production of up to 5.69 kg m^–2^ in outdoor experiments.

In the design of an interfacial solar steam generator, the Janus membrane has been recently proposed for the enhanced device performance. A Janus membrane enhances interfacial solar evaporation processes by improving performance in a number of ways. It is distinguished by having two unique surfaces: one hydrophobic and one hydrophilic. Because the hydrophobic side of the membrane absorbs solar energy and produces high temperatures, the membrane creates a localized heat build-up effect that speeds up the evaporation of water molecules. Furthermore, the bifacial aspect of the membrane enhances the effectiveness of heat transmission by effectively transporting absorbed heat to the water’s surface. The hydrophobic side of the Janus structure blocks the flow of salt ions and other impurities, acting as a barrier against pollutants at the same time and guaranteeing long-term efficacy. For example, by altering polydopamine (PDA) and polydimethylsiloxane (PDMS) on polyurethane sponges, Wang et al.^32^ succeeded in creating Janus evaporators. Under one sun irradiation, Janus evaporators demonstrated great solar energy conversion (90%) and long-term, superior salt resistance in addition to efficient water evaporation (1.26 kg m^–2^ h^–1^). Wang et al.^33^ developed a Janus aerogel by spraying hydrophobic PDMS over a hydrophilic substrate. This resulted in an exceptional 1.83 kg m^–2^ h^–1^ evaporation rate in pure water, and a maximum daily freshwater output of 8.1 kg m^–2^ outdoors.

**Figure 1:**
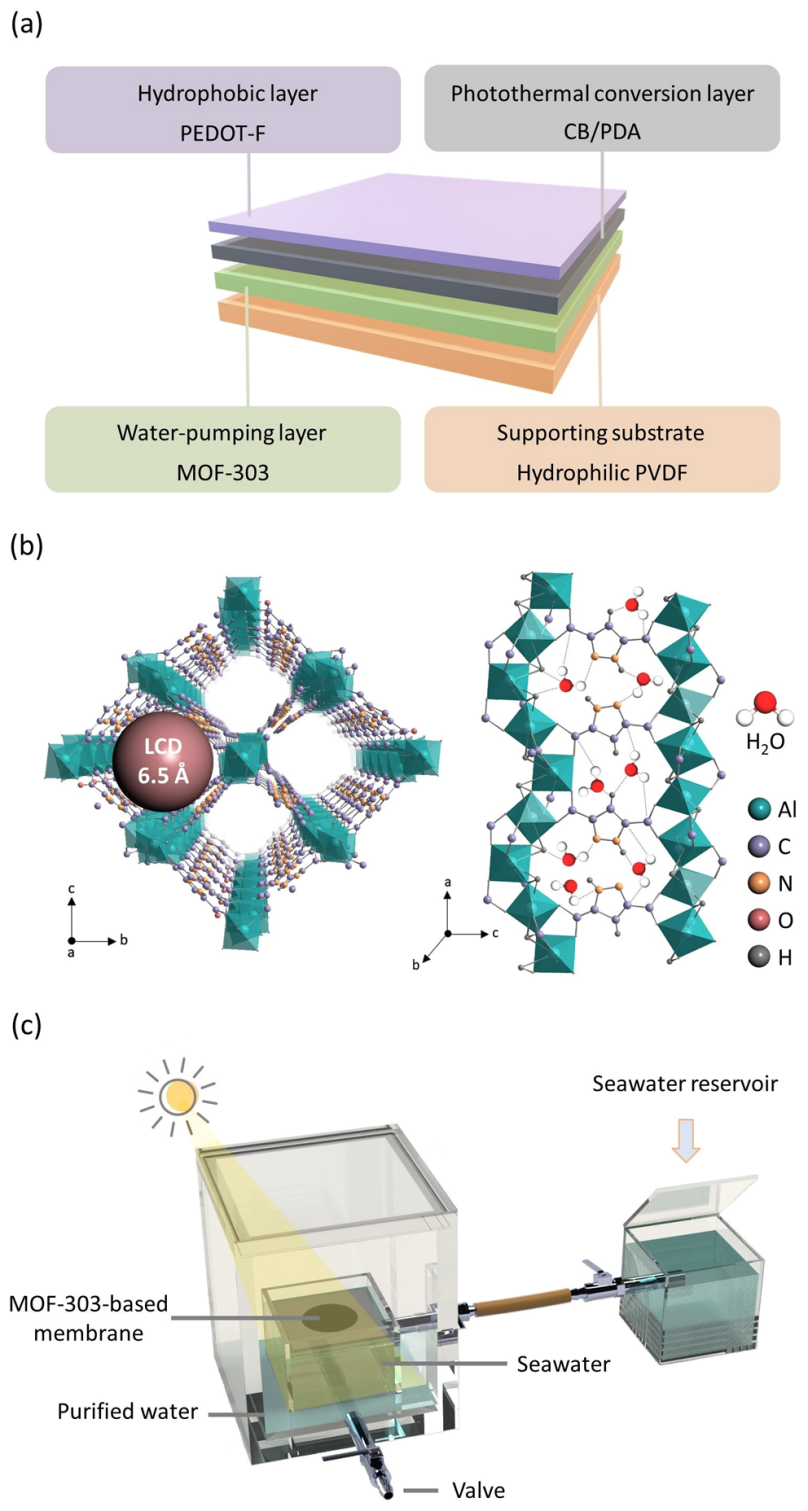
Illustrations of (a) the multilayer structure of the proposed MOF-based membrane for solar steam generation, (b) the crystal structure of MOF-303, showcasing 1D channels with a large cavity diameter of 6.5 Å, and (c) the device for solar steam harvesting.

In this work, we proposed a MOF-based Janus membrane for solar steam generation (Figure 1a,b). MOF-303, with its 1D channels, was chosen as the water-absorbing layer due to its largest cavity diameter of 6.5 Å, which is sufficient for water transport. Known for its high hydrophilicity, MOF-303 has been previously applied for pervaporation, demonstrating high water/ethanol selectivity.^26,27^ Here, we deposited MOF-303 on various substrates to optimize solar steam generation performance, finding that the polyvinylidene difluoride (PVDF) substrate demonstrated the highest efficiency. Materials for solar-to-thermal energy conversion, including carbon black (CB), PDA, and PEDOT-F, were applied to the surface of the MOF-303 membrane. CB and PDA have previously been reported to exhibit excellent photothermal conversion efficiency, reaching up to about 80%^34^ and 67%^35^ respectively. Notably, PEDOT-F not only enhances the efficiency of solar-to-thermal energy conversion but also serves as a hydrophobic layer. Combined with the hydrophilic MOF-303 at the bottom, this creates a Janus membrane. The hydrophobic top layer is designed to prevent salt recrystallization during practical seawater operation. Characterization using Raman spectroscopy and differential scanning calorimetry was conducted to investigate the nanoconfinement effect of MOF-303 on water evaporation enthalpy. We prepared a device (Figure 1c) to measure the evaporation flux for solar steam generation and a separation device for liquid water harvesting. In this setup, the MOF-303-based membrane is placed in the inner tank for water evaporation, while the solar steam is condensed in the outer tank as purified water. Detailed analysis using finite element methods was performed to understand the temperature profiles within the multilayer MOF-based membrane devices.

## 2 Experimental Section

### 2.1 Chemicals and Materials

The chemicals used in this work included aluminum chloride hexahydrate (AlCl_3_·6H_2_O, 99%, Sigma-Aldrich), 3,5-pyrazoledicarboxylic acid monohydrate (H_3_PDC·H_2_O, 97%, Acros Organics), urea (J.T.Baker), sodium chloride (Fluka), ethanol (≥99.8%, Honeywell), methanol (≥99.8%, Honeywell), dichloromethane (≥99.9%, Honeywell), Iron(III) chloride (97%, Sigma-Aldrich), dopamine hydrochloride (Sigma-Aldrich), carbon black (The Far Eastern), hydroxymethyl EDOT (Sigma-Aldrich), 4,4,5,5,6,6,7,7,8,8,9,9,9-tridecafluorononanoic acid (Sigma-Aldrich), 4-dimethylaminopyridine (Sigma-Aldrich), 1-ethyl-3-(3-dimethylaminopropyl) carbodiimide hydrochloride (Sigma-Aldrich). All reagents were used without further purification. The hydrophilic polyvinylidene difluoride (PVDF) membrane filters, featuring pore sizes of 0.45, 0.8, and 5 μm, were acquired from Toson Technology. These filters are sized at 47 mm in diameter with a thickness of 0.2 mm. The deionized water (DI water) was obtained from the ELGA VEOLIA PURELAB ultrapure water system.

### 2.2 Synthesis of MOF-303 Powder

We began by preparing a urea solution, dissolving 3.9 g of urea in 30 g of DI water. Separately, we dissolved 0.722 g of AlCl_3_·6H_2_O and 0.521 g of H_3_PDC·H_2_O in 100 mL of DI water. The aqueous solution was then combined with the urea solution, totaling a volume of 2.65 mL. This mixture underwent reflux at 110 °C for 16 h under continuous agitation, resulting in the formation of MOF-303 crystals in the solution. The obtained MOF-303 powder was collected via vacuum filtration and subsequently dried overnight at 100 °C.

### 2.3 Synthesis of MOF-303 Membranes on PVDF

MOF-303 membranes were synthesized on PVDF substrates (see Figure S1a) utilizing the seeded growth method, a technique commonly employed for MOF membrane synthesis on ceramic substrates.^30,31^ Initially, 0.050 g of MOF-303 powder was dispersed in 10 g of DI water and sonicated for 1 h to form a suspension. Approximately 3 mL of this suspension was spin-coated onto a PVDF substrate at 3000 rpm for 30 s using a Laurell spin-coater (Model-WS-650MZ-23NPPB). The seeded substrate was then baked at 50 °C for 20 min. This seeding process was repeated thrice, followed by overnight drying at 50 °C to prepare for the secondary growth.

To maintain the flatness of the PVDF substrate, a Teflon holder was employed for fixation (Figure S1b). The substrate within the holder was positioned in a 500-mL flask containing the synthesis solution for the secondary growth (Figure S1c). This solution comprised 0.722 g of AlCl_3_·6H_2_O, 0.521 g of H_3_PDC·H_2_O, 100 mL of DI water, and the urea solution (2.65 mL) as prepared earlier. The secondary growth of the MOF-303 membrane was carried out at 110 °C for 16 h. For a 5 μm-thick MOF-303 membrane, the seeded PVDF substrate faced downward at the bottom of the flask during growth. For a 60 μm-thick membrane, the seeded substrate faced upward within the flask. For a 100 μm-thick membrane, the seeded substrate faced upward in the flask, followed by a tertiary growth using the same protocol with a fresh synthesis solution. The resulting membrane underwent rinsing with DI water to remove surface particulates and was then dried at 70 °C before utilization.

### 2.4 Deposition of CB and PDA on the MOF-303 Membrane

Carbon black (CB) and polydopamine (PDA) were deposited on the MOF-303 membrane for the conversion of solar energy into thermal energy. The CB solution was prepared by dispersing 0.05 g of CB in 10 g of ethanol. Subsequently, 2 mL of the CB solution was spray-coated onto the MOF-303 membrane using an airbrush. The membrane, postcoating with the CB layer, was dried at 50 °C for 20 min. To form a PDA layer on the MOF-303 membrane with a layer of CB, we first dissolved 0.08 g of dopamine (DA) in a mixed solvent comprising 40 mL of 70 wt % methanol and 0.8 mL of 0.1 M NaOH. The solution was transferred to a 150 mL glass crystal dish. The MOF-303 membrane deposited on the PVDF substrate sat on the DA solution, with the MOF-303 facing down, for 24 h for the polymerization of DA to form PDA. Following PDA layer growth, the membrane was rinsed with methanol to eliminate particulates on the surface and then dried at 50 °C for 20 min.

### 2.5 Deposition of PEDOT-F on the MOF-303 Membrane

Following the deposition of CB and PDA on the MOF-303 membrane, our next step is to apply a layer of perfluoro-functionalized poly(3,4-ethylenedioxythiophene) (PEDOT-F) on top. Initially, we synthesized perfluoro-functionalized EDOT (EDOT-F), the precursor of PEDOT-F, using a modified version of a previously reported method.^36^ In the synthesis, 440.8 mg of EDOT-OH, 980 mg of 4,4,5,5,6,6,7,7,8,8,9,9,9-tridecafluorononanoic acid, and 590 mg of 4-dimethylaminopyridine were dissolved in 25 mL of dry dichloromethane (DCM). Separately, 590 mg of 1-ethyl-3-(3-dimethylaminopropyl) carbodiimide hydrochloride (EDAC) was dissolved in 5 mL of dry DCM. The EDAC solution was gradually added to the EDOT-OH solution, and the mixture was left to react for 12 h under a N_2_ atmosphere. The resulting product was extracted with a saturated NaCl solution, and the combined organic phase was dried with MgSO_4_. The solvent was then removed via rotary evaporation, and the product underwent purification by flash chromatography (hexane/DCM = 1/3), yielding 800 mg of colorless oil. This oil was stored in a refrigerator at −20 °C, where it solidified into a yellow solid.

To prepare PEDOT-F, we initially dissolved 0.0245 g of EDOT-F in 5 mL of DCM and subjected it to sonication for 10 s. Concurrently, an oxidant solution was prepared by dissolving 0.0162 g of FeCl_3_ in 10 mL of methanol, followed by sonication for 10 s. Next, 0.5 mL of the oxidant solution was spray-coated onto the MOF-303 membrane, which was then dried at 50 °C for 20 min. Subsequently, either 0.5 or 1 mL of the EDOT-F solution was spray-coated onto the same membrane, depending on the desired thickness of the PEDOT-F layer. It is worth noting that 0.5 mL resulted in a thinner layer compared to the 1 mL solution as per the protocol. The membrane was allowed to sit at 25 °C for 10 min for polymerization. After polymerization, the membrane was rinsed with methanol to remove any surface particulates and then dried at 50 °C overnight.

### 2.6 Material Characterization

X-ray diffraction (XRD) was performed using a Rigaku SmartLab SE diffractometer with Cu Kα radiation at a wavelength of 1.5418 Å. The powder X-ray diffraction (PXRD) patterns were acquired from 5 to 40° 2θ with a step size of 0.02° 2θ. The scanning rate was set to be 8° 2θ per minute. Grazing-incidence X-ray diffraction (GIXRD) analysis of the membranes was performed with the incident X-ray beam angled at 0.5°. A diffraction pattern was acquired from 5 to 40° 2θ with a step size of 0.02° 2θ. The scanning rate was set to be 5° 2θ per min, and the incident angle of X-ray was set to be 0.5°.

The morphology of the membranes was investigated using a Hitachi S4800 field emission scanning electron microscope (FE-SEM). The FE-SEM was operated at an acceleration voltage of 10 keV. Before imaging, a thin layer of gold was deposited on the samples via sputtering deposition at a current of 35 mA for 30 s.

Thermogravimetric analysis (TGA) was executed using the TA Instruments SDT 650 system. The heating procedure was programmed to range from 40 to 600 °C, at a steady heating rate of 10 °C min^–1^. The entire measurement was carried out under an airflow at a flow rate of 100 mL min^–1^.

X-ray photoelectron spectroscopy (XPS) was performed using a Thermo Scientific Nexsa G2 system equipped with a monochromatic Al Kα source (1486.6 eV). The instrument was operated under a vacuum of about 10^–8^ mbar within the analyzer chamber.

Differential scanning calorimetry (DSC) measurements were conducted in TA Instruments DSC 25. In a typical measurement, around 15 mg of sample was encapsulated in a 40 μL aluminum pan. Heating and cooling were performed at the rate of 8 °C min^–1^ over a temperature range of – 10 to 200 °C, under a nitrogen gas flow rate of 50 mL min^–1^. Data analysis was carried out using TA Universal Analysis software.

Raman spectra were collected using a Andor Kymera 193i spectrometer under ambient conditions, with excitation at a wavelength of 532 nm.

Nitrogen adsorption isotherms at 77 K were obtained using an Anton Paar Autosorb 6100 system. Approximately 0.1 g of powdered sample was placed into a sample tube. Prior to measurement, the sample underwent degasification under vacuum at 120 °C overnight. Pore size distributions were derived from the raw isotherm data using the Density Functional Theory (DFT) method for calculation.

Inductively coupled plasma optical emission spectrometer (ICP-OES) measurements were conducted for the measurements of ions in the seawater using a Thermo Fisher Scientific iCAP PRO.

### 2.7 Evaluation of Water Evaporation from the Membrane

The membrane subject to the test for the water evaporation was affixed as illustrated in Figure S2a. We use a circular aluminum tape with a diameter of approximately 4 cm, cutting a hole in the middle with a diameter of about 2 cm. The tape is affixed to the membrane, and the junction is sealed with epoxy to prevent the leakage of liquid water. The masked membrane was then placed in a 100 mL beaker containing 50 mL of DI water or 3.5 wt % of NaCl in water, allowing the membrane to float on the water surface.

The beaker with the membrane was placed on an electronic balance (Shimadzu ATX224) for monitoring the mass change caused by water evaporation as illustrated in Figure S2b. A xenon light source (ALS-300 universal light source) was used as a solar simulator. The solar simulator was placed 20 cm above the membrane, and shed light on the membrane device with one sun illumination. A typical test was conducted for approximately 40 min under ambient conditions. The evaporation flux and energy conversion efficiency were calculated via the following equations,
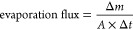
1
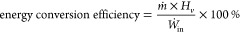
2where Δ*m* represents the mass change of liquid water during the measurement, *A* refers to the effective area of the membrane (value + unit), Δ*t* stands for irradiation time (in h), *ṁ* stands for the measured water evaporation rate that subtracts the background water evaporation rate, which is the water evaporation rate under dark condition (0.22 kg m^–2^ h^–1^), *H*_*v*_ is the enthalpy of vaporization (value + unit), and *Ẇ*_in_ is incident solar radiation density (1 kW m^–2^, i.e., one Sun).

### 2.8 Evaluation of Solar Steam Generation Performance of the Membrane

The homemade facility for evaluating the solar steam generation performance of the MOF membrane is illustrated in Figure S3. This device enables continuous replenishment of feedwater into the reservoir while also collecting fresh liquid water condensed from the evaporated steam. The membrane chamber is connected to a water reservoir to maintain a constant water level during measurements. In a standard test, the membrane chamber is illuminated with a solar simulator at one sun. The feedwater, sourced from tap water with no specified flow rate, is supplied continuously, and the test runs for 3 days. The water recovery of the MOF membrane is defined as the ratio of the water collection rate measured with this device to the water evaporation rate measured from the device described in the previous section.

### 2.9 Simulations of Temperature Distributions within the Device

The finite element method (FEM) was used to calculate the temperature distribution within the membrane device. The FEM was implemented using the commercial software COMSOL Multiphysics 6.0. The simulation geometry mirrors the multilayer structure of the membrane positioned atop liquid water (Figure S4). The equation used to model heat transfer is as follows:
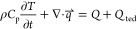
4
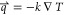
5Where ρ is the density of the medium, *C*_p_ is capacity at constant pressure,  denotes the conductive heat flux, and *k* is the thermal conductivity. The values of the properties in each layer are summarized in Table S1.

The boundary on the top was set as a constant heat flux of 1000 W m^–2^ corresponding to the solar energy input on the surface of the membrane. The side walls were set as thermal insulation to simplify the model. The mesh densities for the finite element method were 10.44 element cm^–2^ for PVDF-CB/PDA membrane on bulk water, 8 element cm^–2^ for PVDF-MOF-303-CB/PDA membrane on bulk water and 14.73 element cm^–2^ for PVDF-MOF-303-CB/PDA-PEDOT-F membrane on bulk water. The initial temperature of the entire system was set at 293.15 K. The time interval for the simulation was set to 0.015 min, and the simulation covered the operation from 0 to 15 min.

## 3 Results and Discussion

### 3.1 Material Characterization of MOF-303

The X-ray diffraction (XRD) analysis of MOF-303 powder and MOF-303 membrane samples is summarized in Figure S5. The XRD pattern of the as-synthesized MOF-303 powder closely matches the simulated pattern from the crystal information file obtained from the Cambridge Crystallographic Data Centre (CCDC) database (CCDC 2078717). The XRD pattern of the MOF-303 membrane grown on the PVDF substrate shows patterns from both the PVDF phase and the MOF-303 phase. However, the peaks attributed to the MOF-303 phase are broader than those in the pattern of the as-synthesized MOF-303 powder. This broadening may be due to the rippled surface of the flexible PVDF substrate, which prevents the MOF-303 membrane from being perfectly flat. To address this issue, we scraped the MOF-303 membrane from the substrate and performed powder XRD. The powder XRD pattern of the scraped MOF-303 closely resembles that of the as-synthesized powder sample without distinct peak broadening. This finding suggests that the MOF-303 membrane grown on PVDF substrates possesses good crystallinity.

The as-synthesized MOF-303 powder was subjected to thermogravimetric analysis (TGA), and the results are shown in Figure S6. A 20% water loss was observed, indicating the high hydrophilicity of the material. The MOF-303 powder remained stable up to approximately 400 °C, demonstrating good thermal stability, which is crucial for solar steam generation applications. Nitrogen adsorption at 77 K was conducted to investigate the porosity of the as-synthesized MOF-303 powder. The adsorption uptake, shown in Figure S7a, was around 230 cm^3^ STP g^–1^, consistent with previously reported values.^37,38^ The pore size distribution, derived from the raw adsorption isotherm using a density functional theory (DFT) based model, is shown in Figure S7b. The pores are in the range of 8 to 10 Å.

### 3.2 Properties of MOF-303 Membranes

We prepared various MOF-303 membranes on the PVDF substrate for subsequent materials. The membrane thickness was adjusted during the secondary growth by controlling experimental conditions. For the 10-μm MOF-303 membrane, the membrane faced downward at the bottom of the flask. For a 60 μm-thick membrane, the seeded substrate faced upward in the flask. For a 100 μm-thick membrane, the seeded substrate faced upward in the flask, followed by a tertiary growth using the same protocol with a fresh synthesis solution. The SEM images of the bare PVDF substrate and MOF-303 membranes with different thicknesses (10, 60, and 100 μm) are summarized in Figure S8a–d. The SEM images show that the bare PVDF substrates have voids in the range of tens of micrometers. After the deposition of MOF-303, a dense layer formed on the substrate, and these voids were no longer visible from the top view. To improve solar energy conversion efficiency, a layer of CB/PDA was deposited on the MOF-303 membrane with 60 μm in thickness, and the sample is named MOF-303-CB/PDA. An additional layer of PEDOT-F was then deposited to enhance surface hydrophobicity, and this sample is named MOF-303-CB/PDA-PEDOT-F. The SEM images of MOF-303-CB/PDA-PEDOT-F are shown in Figure S8e.

**Figure 2:**
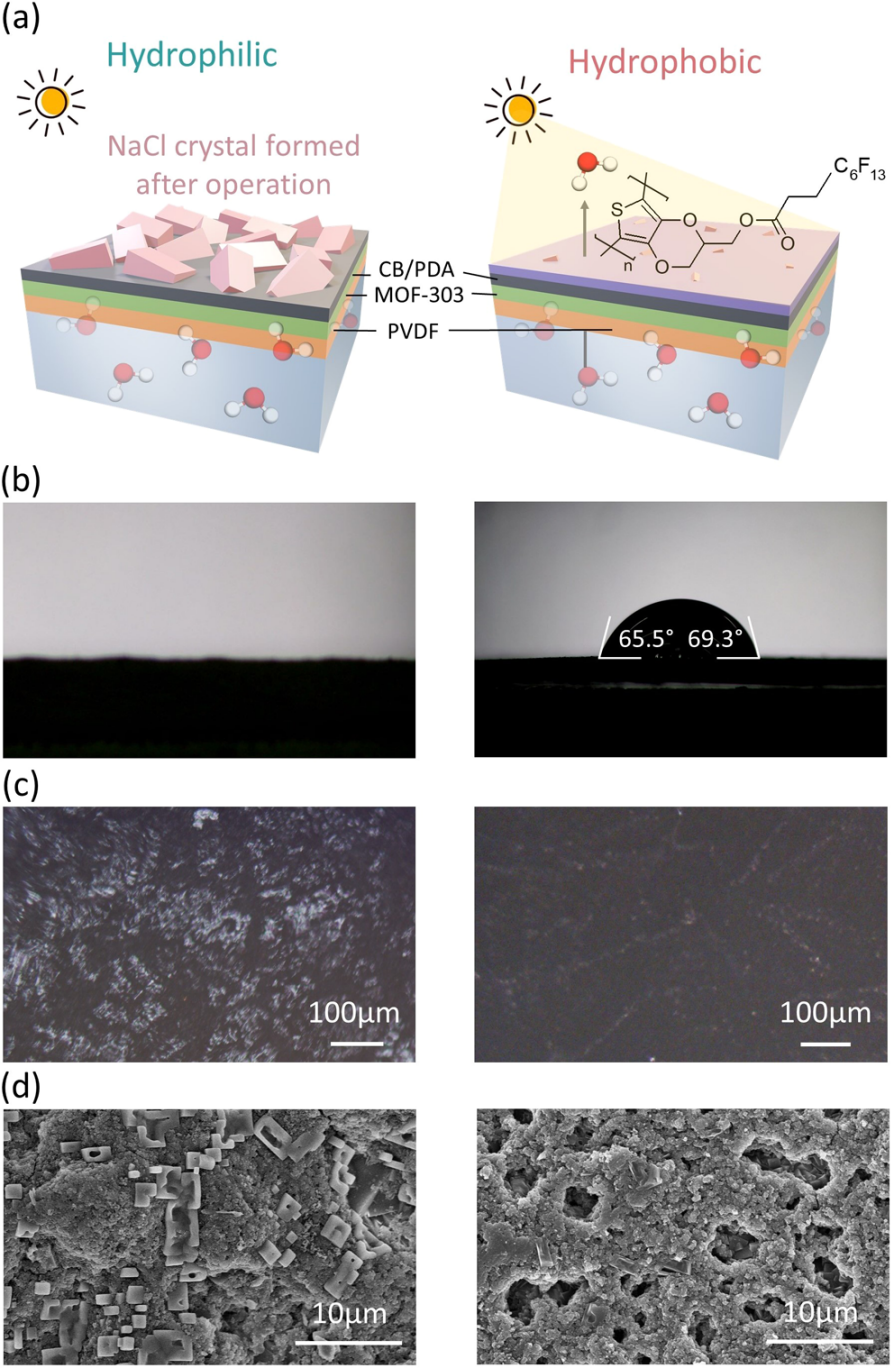
(a) Illustration of NaCl crystallization on the hydrophilic surface of the MOF-303-CB/PDA membrane after operating solar steam generation with a 3.5% NaCl aqueous solution (left). This issue was mitigated by the deposition of hydrophobic PEDOT-F (right). (b) Water contact angle measurements on the MOF-303-CB/PDA and MOF-303-CB/PDA-PEDOT-F membranes. (c) Optical and (d) and SEM images, respectively, of the top surface of MOF-303-CB/PDA (left) and MOF-303-CB/PDA-PEDOT-F (right) membranes.

X-ray photoelectron spectroscopy (XPS) was used to analyze the multilayer composition of the MOF-303 membranes. The XPS spectrum of the bare PVDF substrate, shown in Figure S9a, is dominated by signals of fluorine and carbon. After depositing a MOF-303 layer on PVDF, the fluorine signals were barely visible (Figure S9b). Instead, peaks attributed to aluminum and nitrogen appeared, corresponding to the composition of MOF-303. The XPS spectra of the MOF-303-CB/PDA-PEDOT-F membrane before and after etching for 5 s are shown in Figures S9c and S9d, respectively. Compared to the MOF-303 membrane, the aluminum peak was barely visible in the pristine MOF-303-CB/PDA-PEDOT-F, indicating that the MOF-303 layer is underneath the CB/PDA and PEDOT-F layers, making it undetectable by surface characterization. After etching with an argon ion beam for 5 s, the fluorine peak decreased, suggesting that the top layer of MOF-303-CB/PDA-PEDOT-F was successfully covered by PEDOT-F. UV–vis spectroscopy was also conducted on the multilayer composition of the MOF-303 membranes (Figure S10). While the MOF-303 layer did not exhibit strong absorption, the CB, PDA, and PEDOT-F layers all displayed strong absorption across the UV–visible spectrum. The measurements of water contact angle are summarized in Figure S11. The water contact angles for the bare PVDF substrate, and MOF-303 and MOF-303-CB/PDA membrane on PVDF substrates were difficult to measure. Due to the high surface hydrophilicity and intrinsic pores within the MOF-303 and PVDF substrate, a water droplet quickly penetrated the membrane, making the contact angle unmeasurable. However, after the deposition of PEDOT-F, the water contact angle became 69°, indicating a decrease in surface hydrophilicity due to the hydrophobic nature of PEDOT-F. The hydrophobicity of the membrane device’s top surface is critical for desalination applications. We tested two different membrane samples for solar steam generation for 1 h using an aqueous solution containing 3.5 wt % NaCl. Following the operation, we observed the recrystallization of NaCl on the top surface of the MOF-303-CB/PDA membrane, which is hydrophilic (Figure 2). In contrast, the MOF-303-CB/PDA-PEDOT-F membrane showed reduced salt recrystallization. The hydrophobic surface created by the PEDOT-F coating increased the water contact angle from 0 to 69°, thereby preventing salt recrystallization.

**Figure 3:**
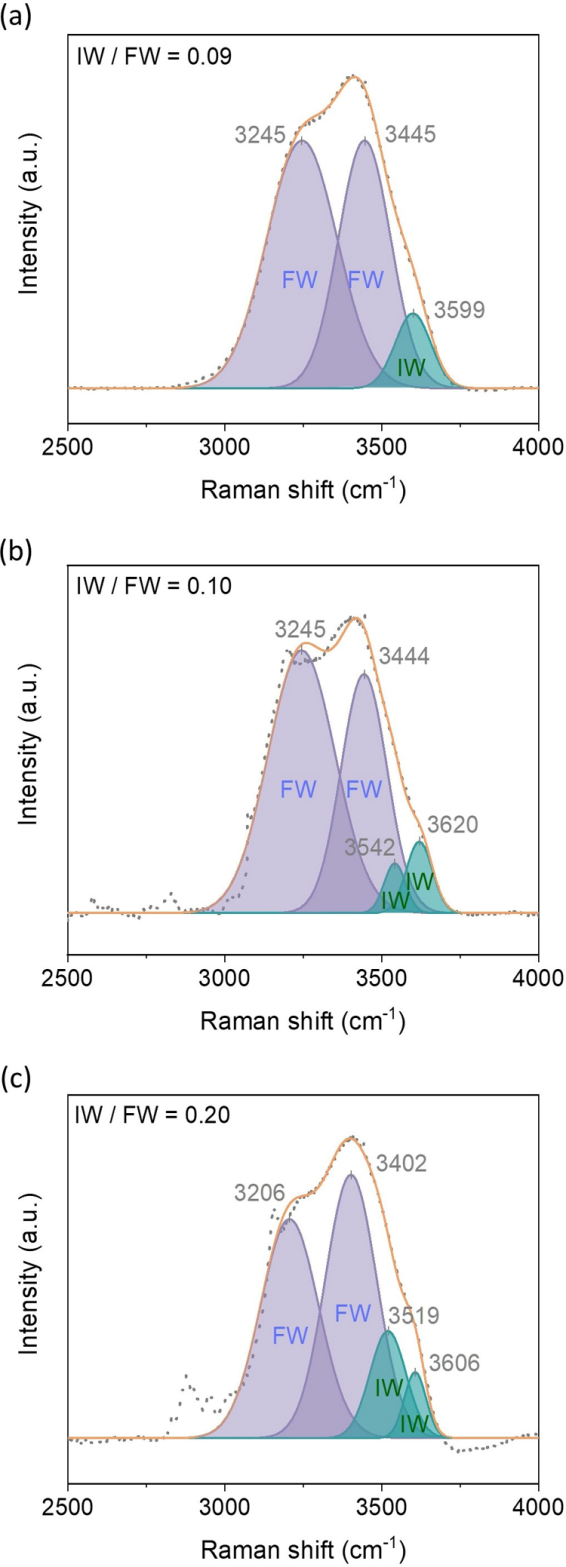
Raman spectra of (a) liquid water, (b) water in the PVDF substrate, and (c) water in the MOF-303 membrane. The spectra highlight the presence of intermediate water (IW) and free water (FW). The IW/FW ratios for liquid water, water in the PVDF substrate, and water in the MOF-303 membrane are 0.09, 0.1, and 0.2, respectively.

We utilized Raman spectroscopy to investigate the intermolecular interactions of water in MOF-303. The spectral region from 2700 to 3800 cm^–1^ corresponds to various −OH stretching modes of water molecules.^39^ Previous studies^40−42^ have distinguished two types of water in a Raman spectrum: free water (FW) and intermediate water (IW). FW refers to water molecules that are not strongly bound to surfaces or other molecules, behaving similarly to bulk water. In contrast, IW represents a state of water that exists between strongly adsorbed water and FW, partially influenced by the nanoconfinement. FW is shown in the spectral region of 3200–3500 cm^–1^, whereas IW is shown in the 3500–3700 cm^–1^ region. Typically, the formation of IW in a porous medium reduces the enthalpy of water evaporation. To obtain the Raman spectra of water in the membrane samples, 100 μL of water was placed on a piece of the membrane sample (0.1 g) before analysis. The Raman spectra are summarized in Figure 3. The liquid water and water in the PVDF substrate showed similar IW/FW ratios (0.09 and 0.10, respectively), whereas the IW/FW ratio measured for MOF-303 increased to 0.20. Considering the hydrophilic interior surface of MOF-303,^26,27^ it is likely that it facilitates the formation of IW within the micropores.

**Figure 4:**
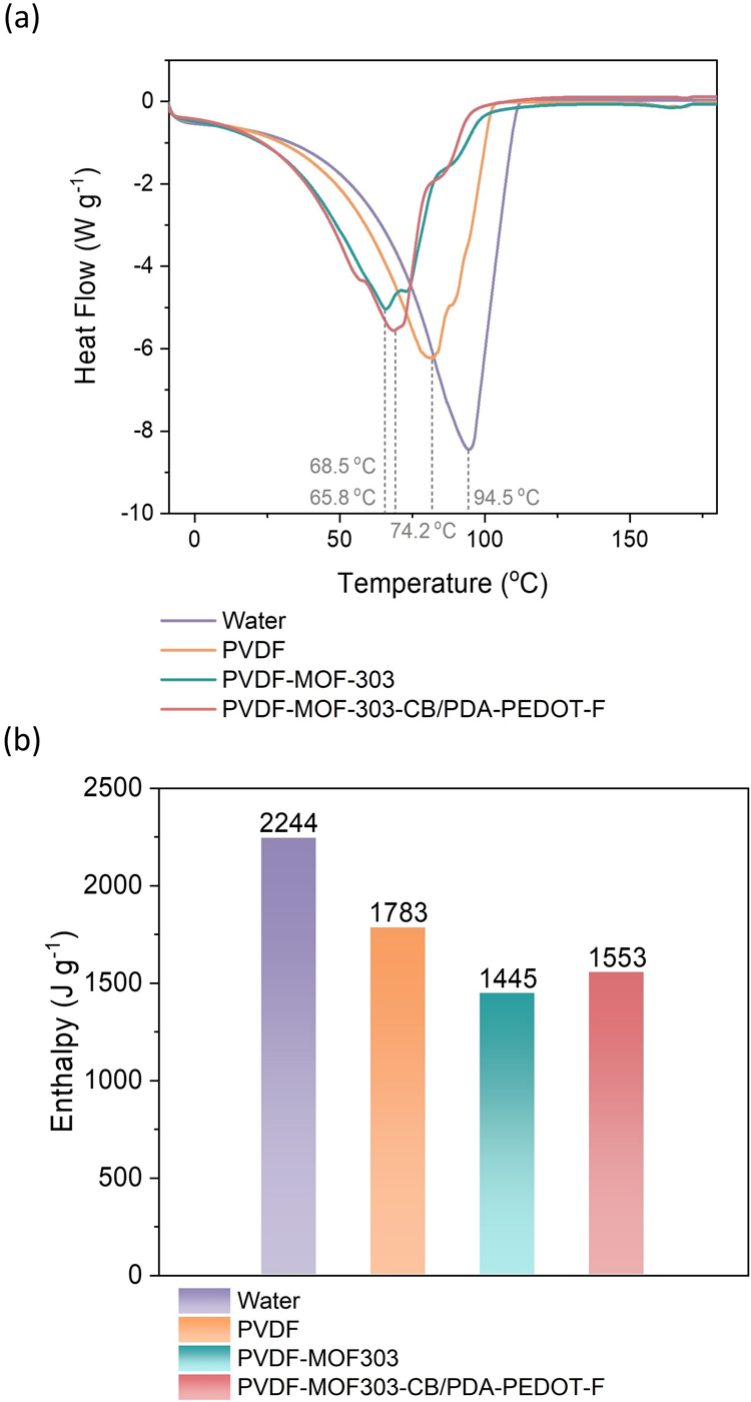
(a) DSC measurements and (b) derived enthalpy for water evaporation of free liquid water, water in the PVDF, and water in the MOF-303 membrane, and MOF-303 membrane with photothermal layer.

Differential scanning calorimetry (DSC) was performed to understand the vaporization behavior of water from the membranes, with the results summarized in Figure 4. For a free water droplet, the vaporization curve showed a maximum value at 94.5 °C. The enthalpy of vaporization for the free water droplet was calculated as 2244.3 J g^–1^, consistent with the theoretical value of 2444.7 J g^–1^.^43^ For the investigation of water vaporization from the membrane samples, 100 μL water was dropped onto samples weighing 0.1 g. After a 10-min waiting period, the samples were cut into small pieces, and approximately 20 mg of these pieces were placed into a Tzero aluminum pan for measurement. For the water in the PVDF substrate and the water in MOF-303 grown on PVDF, the DSC curves for water vaporization peaked at 74.2 and 65.8 °C, respectively. The lowered evaporation temperatures indicate that water adsorbed in PVDF or MOF-303 requires less heat for phase transition to vapor compared to liquid water. Additionally, the enthalpies associated with water vaporization were found to be 1783.3 J g^–1^ for PVDF and 1445.2 J g^–1^ for MOF-303, both significantly lower than that of free water. These results suggest that MOF-303 grown on the PVDF substrate can significantly reduce the energy required for the vaporization of water. The functional groups from the photothermal materials could influence water adsorption and evaporation. To investigate this, we conducted DSC measurements on the MOF-303-CB/PDA-PEDOT-F membrane. The results indicate that the MOF membrane, both with and without the photothermal materials, exhibited similar water evaporation temperatures and enthalpies. This experiment demonstrates that the functional groups from the photothermal materials do not play a critical role in this context. Instead, the water evaporation properties are primarily governed by the nanoconfinement within MOF-303.

### 3.3 Water Evaporation and Solar Steam Generation with MOF-303 Membranes

We assessed various parameters to maximize the water evaporation rate through MOF-303 membranes. Initially, we evaluated the water evaporation rate through blank substrates (Figure S12a). Among the eight substrates tested, the hydrophilic PVDF substrate with an average pore size of 5 μm exhibited a significantly higher water evaporation flux. Consequently, the PVDF substrate with a 5-μm pore size was selected for subsequent studies. We also investigated the effects of the thickness of the MOF-303, CB, and PEDOT-F layer on the water evaporation rate (Figure S12b). While increasing the thickness of the photothermal materials can raise the surface temperature, it also increases the mass transfer resistance for water transport. Therefore, there is an optimal thickness for the photothermal materials that balances these factors for effective water harvesting applications. The optimized membrane, comprising a 60-μm MOF-303 layer and a thin layer of PEDOT-F, achieved a water evaporation flux of 2.32 kg h^–1^ m^–2^, significantly surpassing the flux of bare substrate 0.79 kg h^–1^ m^–2^.

The water evaporation flux mentioned above was measured using the device shown in Figure S2, which only investigates water evaporation rather than water harvesting. However, collecting water vapor following evaporation is crucial in the study of membranes for solar steam generation. Designing a device for harvesting liquid water is nontrivial. We proposed three versions of a device designed to harvest liquid water from solar steam generated by a MOF membrane. The evolution of these devices is summarized in Figure S13. Version 1 of the device lacked proper sealing and suffered from severe water vapor leakage. In version 2, a sponge was added between the lid and the box for better sealing, which alleviated but did not completely resolve the leakage issue. Additionally, version 2 was a batch system rather than a continuous flow system, limiting its operation. Version 3, however, was designed as a continuous flow system, allowing for constant water inflow and outflow. An O-ring was added between the lid and the box, providing excellent sealing and minimizing water vapor leakage. For subsequent experiments, version 3 was used for harvesting liquid water from solar steam.

**Figure 5:**
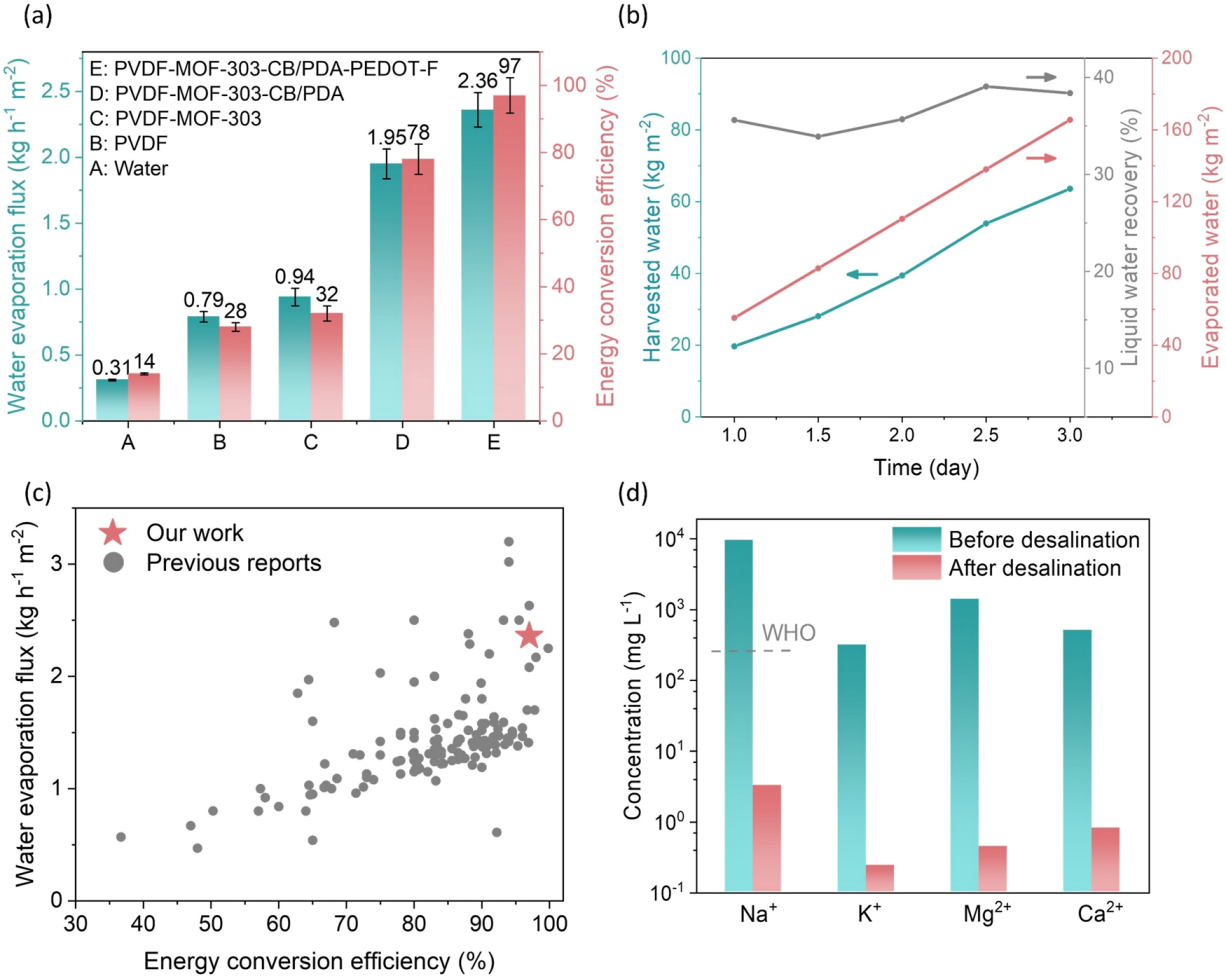
(a) Water evaporation flux and energy conversion efficiency of various membrane samples under one sun irradiation. Deionized water was used for the measurements. (b) Evaporated water, harvested water, and liquid water recovery versus time under one sun irradiation. Deionized water was used for the measurements. (c) Water flux versus energy conversion efficiency of our membrane device (MOF-303-CB/PDA-PEDOT-F) compared to previously reported devices. (d) Ion concentrations of the initial seawater and the seawater after desalination.

Our membrane device for solar steam generation features multiple layers, each serving a specific function. To elucidate the role of each layer, we investigated water evaporation over time for various membrane configurations (Figure 5a). Under one sun irradiation, the water evaporation flux for deionized water was measured at 0.31 kg h^–1^ m^–2^. Using a porous hydrophilic PVDF substrate, the flux increased to 0.79 kg h^–1^ m^–2^, with an energy conversion efficiency of 28%. When a MOF-303 layer was added to the PVDF substrate, the water evaporation flux increased slightly to 0.94 kg h^–1^ m^–2^ and the energy conversion efficiency to 32%, indicating that the MOF-303 layer alone does not significantly enhance solar energy conversion due to its reflective white layer. To further improve the efficiency, we deposited CB/PDA on the MOF-303 membrane, which increased the water evaporation flux to 1.95 kg h^–1^ m^–2^ and the energy conversion efficiency to 78%. The final optimization involved coating a layer of PEDOT-F on top, resulting in an optimized MOF-303-based membrane (MOF-303-CB/PDA-PEDOT-F) that achieved a water evaporation flux of 2.36 kg h^–1^ m^–2^ and a high energy conversion efficiency of 97%. We also evaluated the water evaporation efficiency of the optimized MOF-303-CB/PDA-PEDOT-F membrane by placing the device outdoors and utilizing real sunlight for water evaporation (Figure S14). Between 10 am and 8 pm, with temperatures ranging from a high of 33 to a low of 30 °C, the water evaporation flux was measured to be 5.7 kg h^–1^ m^–2^. The outdoor water evaporation flux was significantly higher than that observed indoors, likely due to the higher ambient temperatures and improved air convection outdoors.

We further evaluated the MOF-303-CB/PDA-PEDOT-F membrane for liquid water harvesting using the version 3 harvesting device (Figure 5b). Due to minor water vapor leakage, the liquid water harvesting rate was approximately one-third of the water evaporation rate, with liquid water recovery ranging from 35 to 40%. Figure 5c summarizes the water evaporation flux versus energy conversion efficiency, comparing our device with 145 previous reports (Table S2). Our device demonstrated good water evaporation flux and excellent energy conversion efficiency (97%) compared to others. We have also compared the device performance for solar steam generation among devices with Janus structures and MOF-based devices (Figure S15). The optimized MOF-303 devices reported in this study demonstrates competitive performance. Additionally, we tested the desalination capability of the MOF-303-CB/PDA-PEDOT-F membrane using real seawater (Figure S16). Ion concentrations were analyzed via ICP-OES, and the conductivity of seawater was measured before and after desalination (Figure 5d). The metal ion concentrations (Na^+^, K^+^, Mg^2+^, and Ca^2+^) after desalination dropped by at least 2 orders of magnitude, meeting WHO regulations for drinking water.

**Figure 6:**
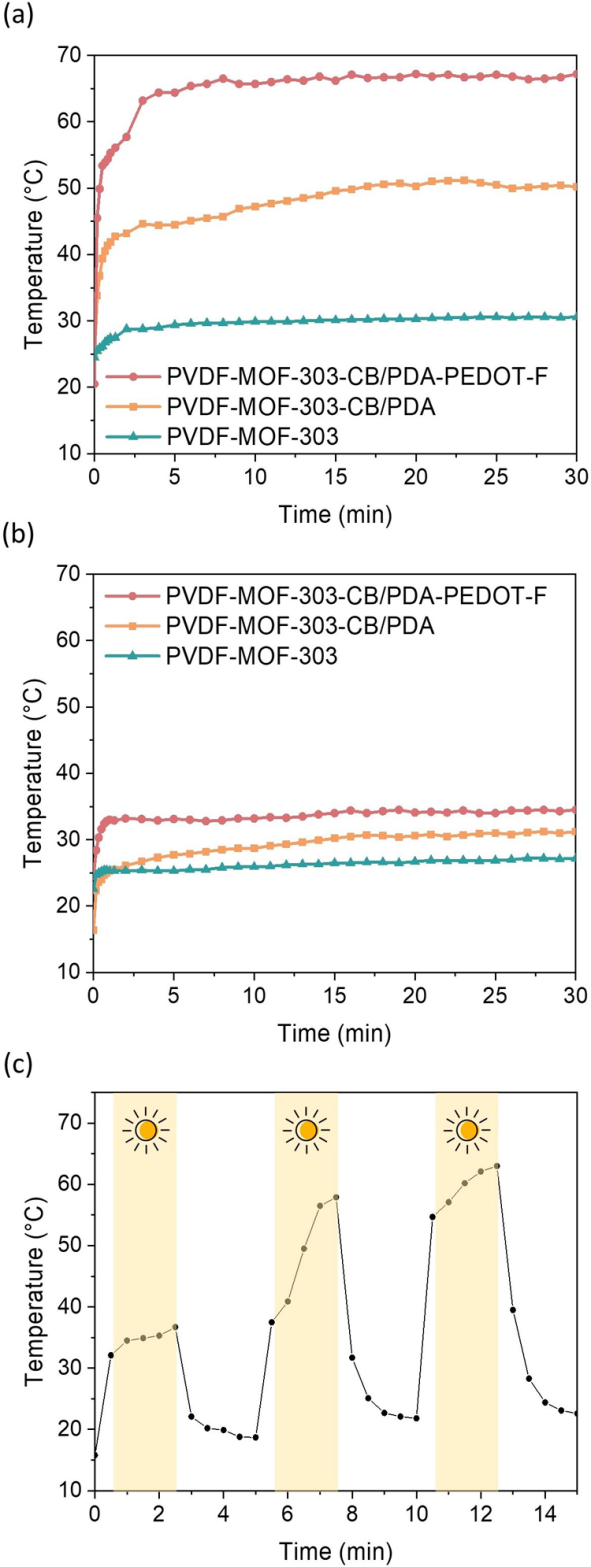
Surface temperature curves measured via an infrared thermometer for samples placed on (a) a lab table and (b) liquid water under one sun irradiation. (c) Surface temperature of PVDF-MOF303-CB/PDA-PEDOT-F membranes on a lab table subjected to an on-and-off test under one sun irradiation.

To further understand the role of the thermal conversion layers, namely CB, PDA, and PEDOT-F, we studied the surface temperature of various membrane samples under one sun irradiation. The surface temperature was measured using an infrared thermometer. The first set of experiments involved placing the samples on a table (Figure 6a), while the second set involved placing the membranes on liquid water (Figure 6b). For the samples placed on the table, the surface temperature of the MOF-303 membrane reached only 30.6 °C under one sun irradiation. In contrast, the surface temperature of the MOF-303-CB/PDA membrane significantly increased to 50.2 °C, and the MOF-303-CB/PDA-PEDOT-F sample further improved to 67.2 °C. This demonstrates the critical role of CB, PDA, and PEDOT-F in converting solar energy to thermal energy. A similar trend was observed in the experiments where the samples were placed on liquid water. The surface temperatures of the MOF-303, MOF-303-CB/PDA, and MOF-303-CB/PDA-PEDOT-F membranes were 27.2, 31.2, and 34.5 °C, respectively. The optimized MOF-303-CB/PDA-PEDOT-F sample was subjected to on-and-off illumination tests (Figure 6c) on the table, revealing that the surface temperature quickly reached 35.3 °C within 2 min

**Figure 7:**
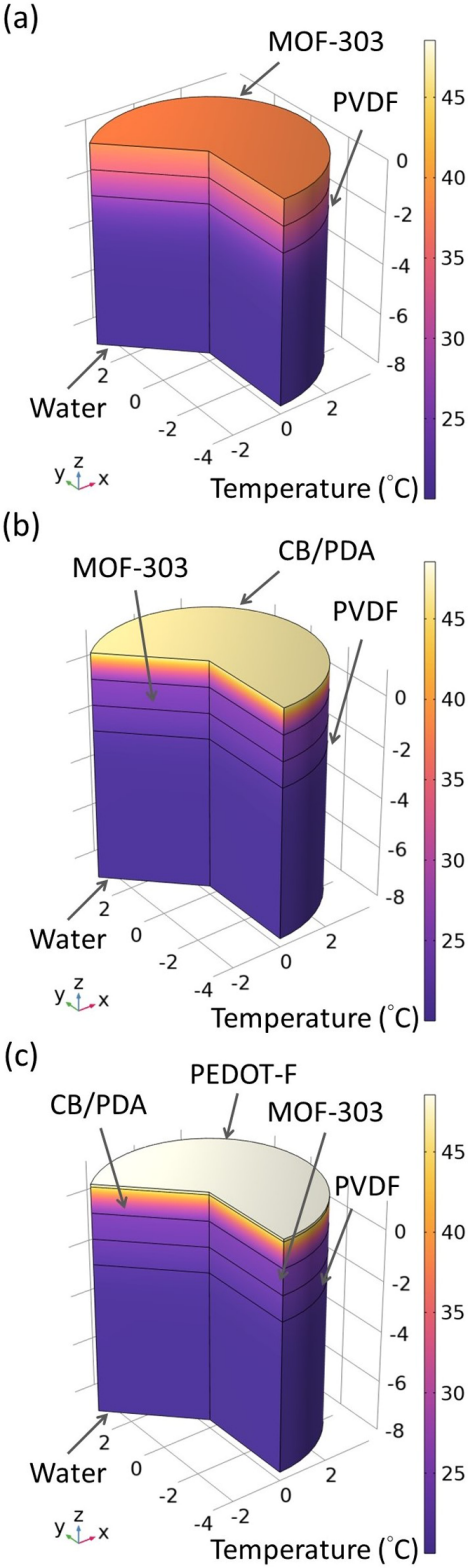
Temperature profiles from FEM-based simulations of (a) PVDF-MOF-303, (b) PVDF-MOF-303-CB/PDA, and (c) PVDF-MOF-303-CB/PDA-PEDOT-F membranes placed on liquid water.

Simulations based on the finite element method (FEM) were performed to investigate the temperature profiles within the multilayer membrane devices, with results summarized in Figure 7. In the simulations, the water temperature was initialized at 20 °C. The temperature profiles of the three samples—MOF-303, MOF-303-CB/PDA, and MOF-303-CB/PDA-PEDOT-F—presented distinct differences. Specifically, the temperature at the top surface of MOF-303-CB/PDA-PEDOT-F reached 47.8 °C, while the MOF-303 surface was at 37.7 °C. Notably, the surface temperatures from the FEM simulations appeared higher than those from the experiments shown in Figure 6. This discrepancy is due to the penetration of infrared thermometer into the membrane samples, resulting in an average temperature readout over hundreds of micrometers in thickness.

## 4 Conclusions

In this work, we proposed a MOF-based Janus membrane for solar steam generation, leveraging the hydrophilic 1D channels of MOF-303 as the water-absorbing layer. The MOF-303, with its largest cavity diameter of 6.5 Å, demonstrated sufficient capacity for water transport. Our deposition of MOF-303 on various substrates aimed to optimize solar steam generation performance. Characterization with Raman spectroscopy reveals that the nanoconfinement within MOF-303 leads to a higher proportion of intermediate water (IW) relative to free water (FW), as indicated by an IW/FW ratio of 0.2, compared to 0.09 in bulk liquid water. Differential scanning calorimetry characterization revealed that the water evaporation enthalpy in MOF-303 decreased to 1445.2 J g^–1^ compared to 2244.3 J g^–1^ for bulk liquid water, indicating the potential of MOF-303 for energy-efficient water evaporation from the feed. We incorporated materials for solar-to-thermal energy conversion, including carbon black (CB), polydopamine (PDA), and PEDOT-F, onto the surface of the MOF-303 membrane. Notably, PEDOT-F enhanced solar-to-thermal energy conversion efficiency and served as a hydrophobic layer. This combination with the hydrophilic MOF-303 created a Janus membrane, designed to prevent salt recrystallization during practical seawater operations.

Under one sun irradiation, the water evaporation flux for deionized water was measured at 0.31 kg h^–1^ m^–2^. Using a porous hydrophilic PVDF substrate, the flux increased to 0.79 kg h^–1^ m^–2^ with an energy conversion efficiency of 28%. Adding a MOF-303 layer to the PVDF substrate slightly increased the water evaporation flux to 0.94 kg h^–1^ m^–2^ and the energy conversion efficiency to 32%, suggesting that the MOF-303 layer alone does not significantly enhance solar energy conversion due to its reflective white layer. To further improve efficiency, we deposited CB/PDA on the MOF-303 membrane, which increased the water evaporation rate to 1.95 kg h^–1^ m^–2^ and the energy conversion efficiency to 78%. The final optimization involved coating a layer of PEDOT-F on top, resulting in an optimized MOF-303-based membrane (MOF-303-CB/PDA-PEDOT-F) that achieved a water evaporation rate of 2.36 kg h^–1^ m^–2^ and a high energy conversion efficiency of 97%.

Additionally, the desalination capability of the MOF-303-CB/PDA-PEDOT-F membrane was demonstrated, with metal ion concentrations (Na^+^, K^+^, Mg^2+^, and Ca^2+^) after desalination dropping by at least 2 orders of magnitude, meeting WHO regulations for drinking water. These results demonstrate that the MOF-303-based Janus membrane holds significant potential for efficient solar steam generation, combining high water evaporation rates with high energy conversion efficiency. The incorporation of additional layers such as CB, PDA, and PEDOT-F has been crucial in optimizing performance, making this approach promising for practical applications in solar steam generation and desalination.

## Supporting Information Available

The Supporting Information is available free of charge. Photographic images of the substrate for membrane deposition, reactor setup for synthesis, membrane masking, and solar steam generation testing setups; illustrations of geometry and mesh setups for FEM simulations; XRD patterns; TGA curve; pore size distribution analysis; SEM images of membranes; XPS and UV–vis spectra; water contact angle measurements; and performance comparisons of solar steam generation devices (PDF)
