# Supplementary material for: MOF-303 with Lowered Water Evaporation Enthalpy for Solar Steam Generation
Source: ACS Appl Mater Interfaces. 2024 Sep 6;16(37):49640–50. doi: 10.1021/acsami.4c10506 (PMC11420869; doi:10.1021/acsami.4c10506)
Supplement: Supplementary file 1 — am4c10506_si_001.pdf [file am4c10506_si_001.pdf]

Supporting Information

for

MOF-303 with Lowered Water Evaporation Enthalpy for Solar Steam Generation

Yi-Hsuan Lin[a] ,Hsun-Hao Lin[b], Yu-Shuo Lee[a], Wen-Yueh Yu[a], Shyh-Chyang Luo[b,*] and Dun-Yen Kang[a,*]

[a]*Department of Chemical Engineering, National Taiwan University, No. 1, Sec. 4, Roosevelt Road, Taipei 106319, Taiwan*

[b]*Department of Materials Science and Engineering, National Taiwan University, No. 1, Sec. 4, Roosevelt Road, Taipei 106319, Taiwan*

Shyh-Chyang Luo *E-mail ：shyhchyang@ntu.edu.tw

Dun-Yen Kang *E-mail: dunyen@ntu.edu.tw

Supporting Figures

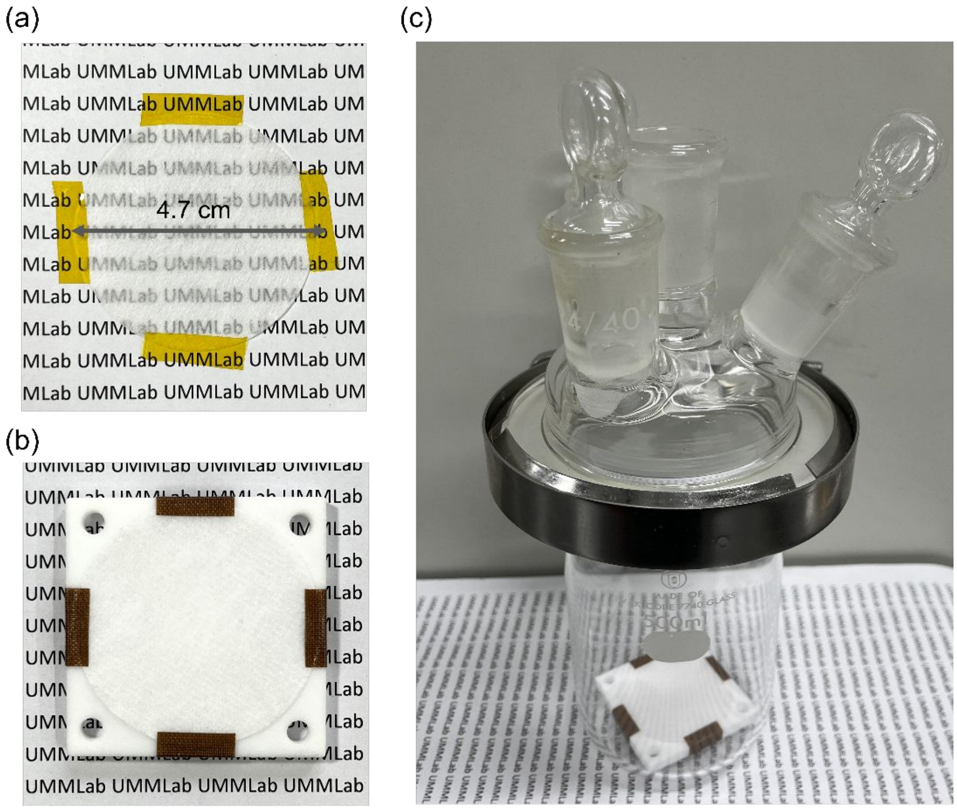

Figure S1. Photographic images of (a) the PVDF substrate, (b) the Teflon holder used to fix the PVDF substrate to prevent bending, and (c) the reactor setup for the synthesis of the MOF-303 membrane.

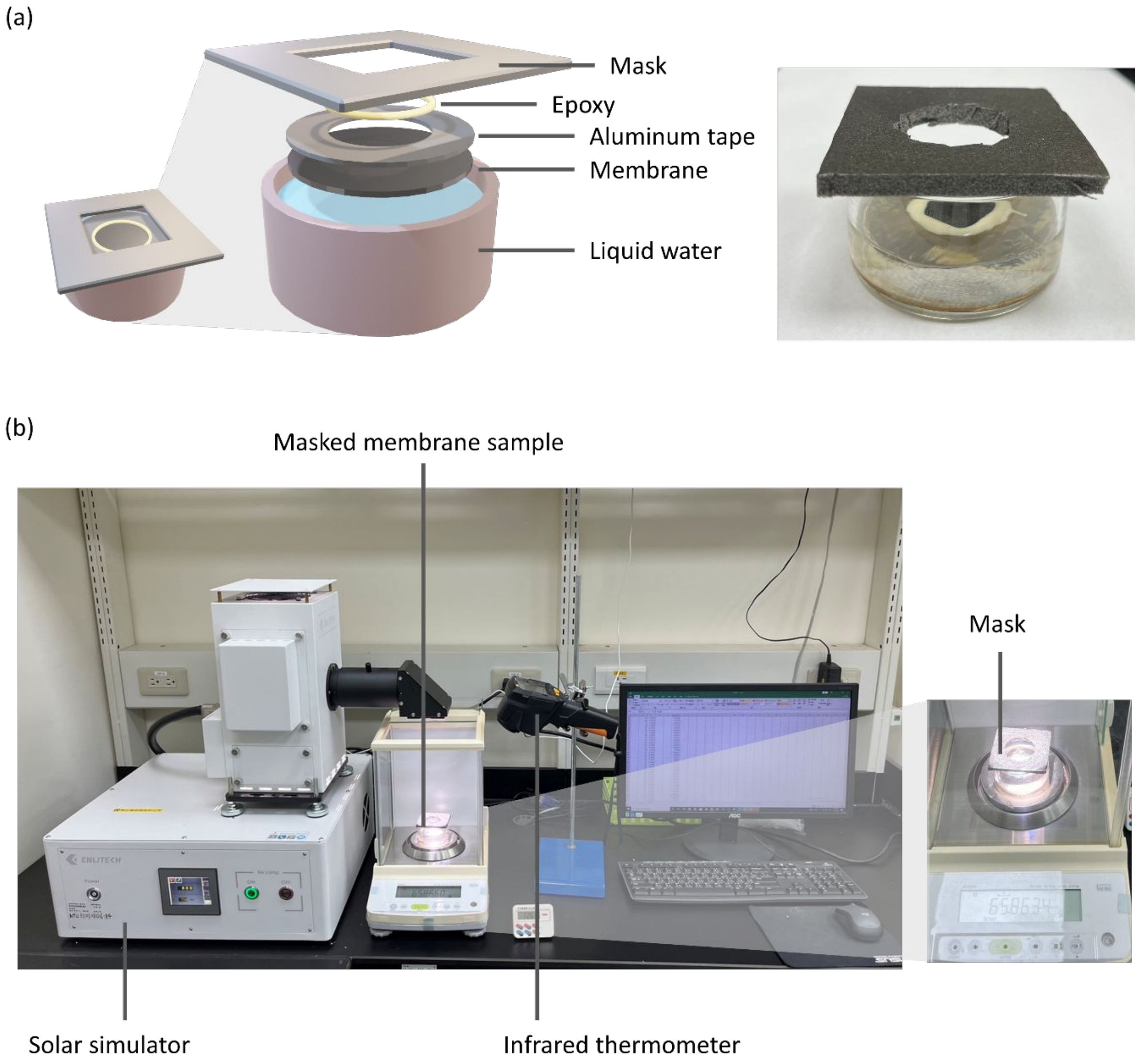

Figure S2. (a) Illustration of the membrane masking method for measuring the water evaporation rate, and (b) photographic image of the setup used for testing solar steam generation, including the solar simulator.

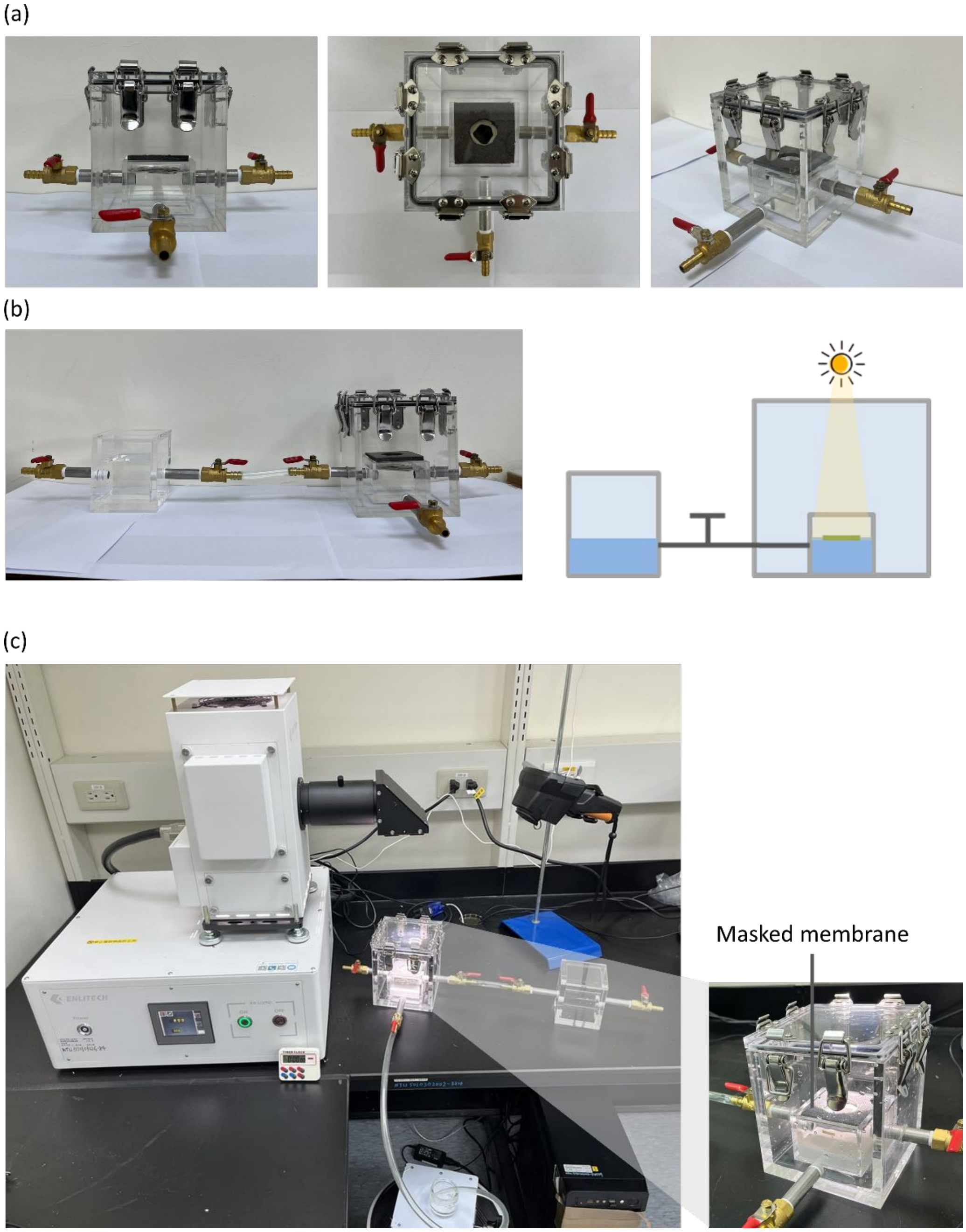

Figure S3. Photographic images of (a) the membrane chamber of the device for harvesting liquid water from solar steam, (b) the membrane chamber connected to a feed water reservoir, shown with a photograph and a schematic illustration, and (c) the entire setup for harvesting liquid water from solar steam.

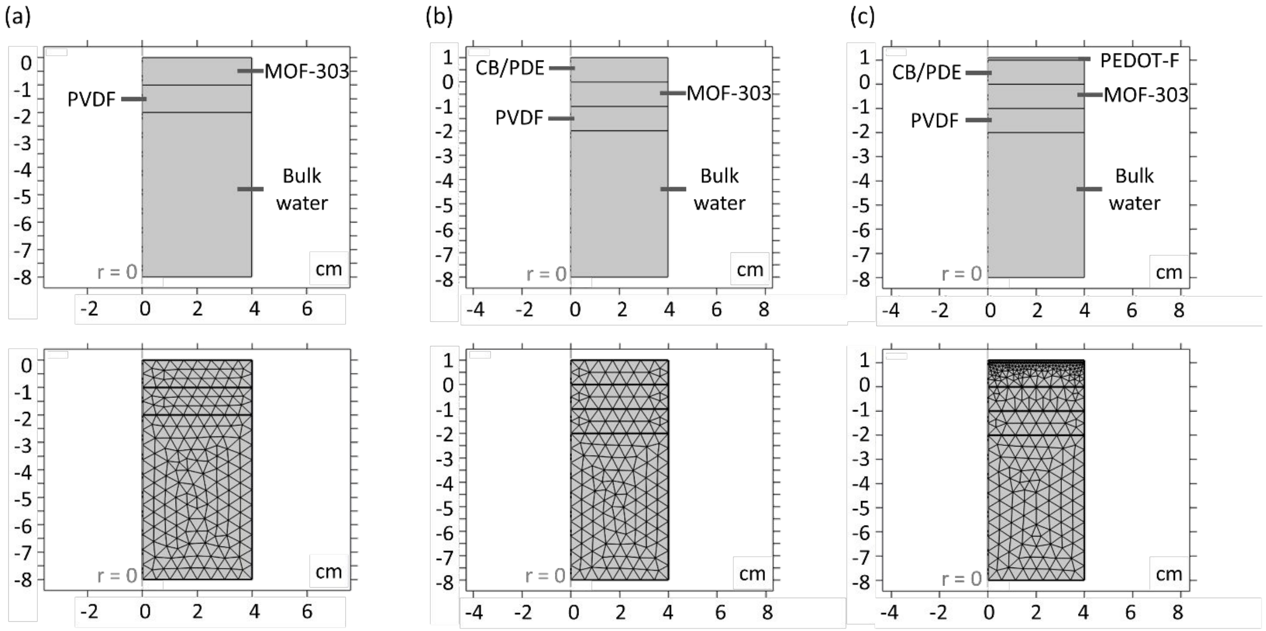

Figure S4. Geometry and mesh setup of various models for simulating temperature profiles using the finite element method: (a) PVDF-MOF-303 membrane, (b) PVDF-MOF-303-CB/PDA membrane, and (c) PVDF-MOF-303-CB/PDA-PEDOT-F membrane on liquid water.

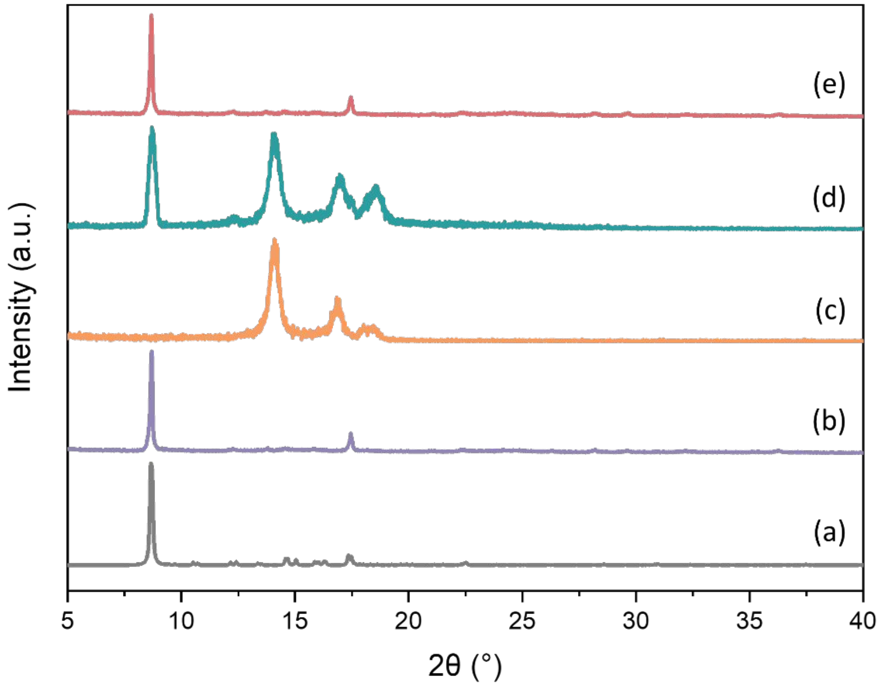

Figure S5. (a) Simulated powder XRD pattern of MOF-303; XRD patterns obtained experimentally for (b) as-synthesized MOF-303 powder, (c) bare PVDF substrate, (d) MOF-303 membrane grown on PVDF, and (e) MOF-303 powder scraped from the membrane grown on the PVDF substrate.

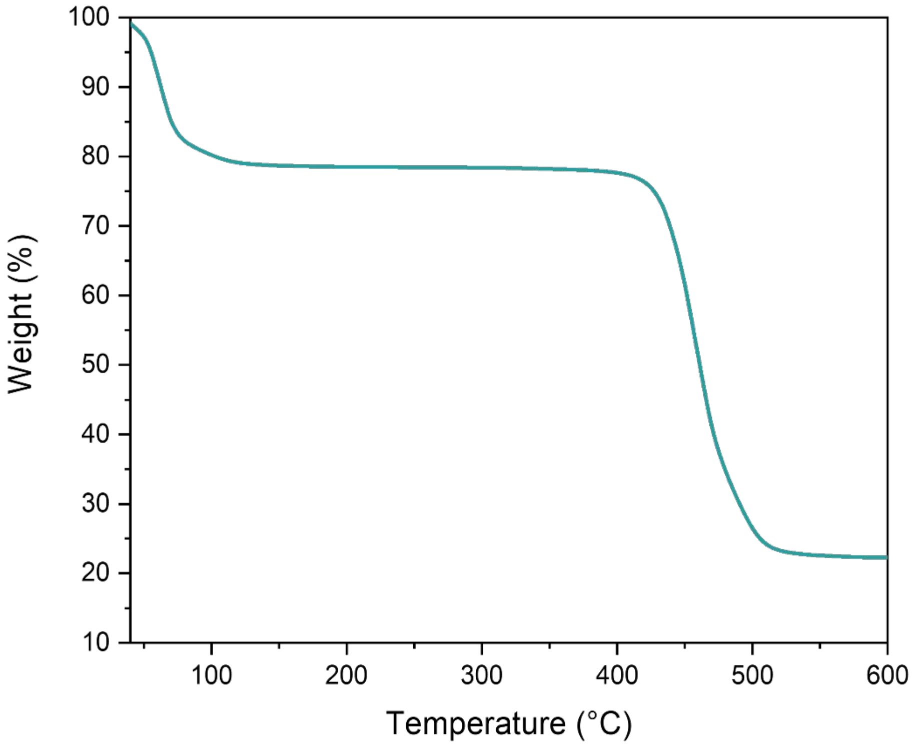

Figure S6. TGA curve of the as-synthesized MOF-303 powder.

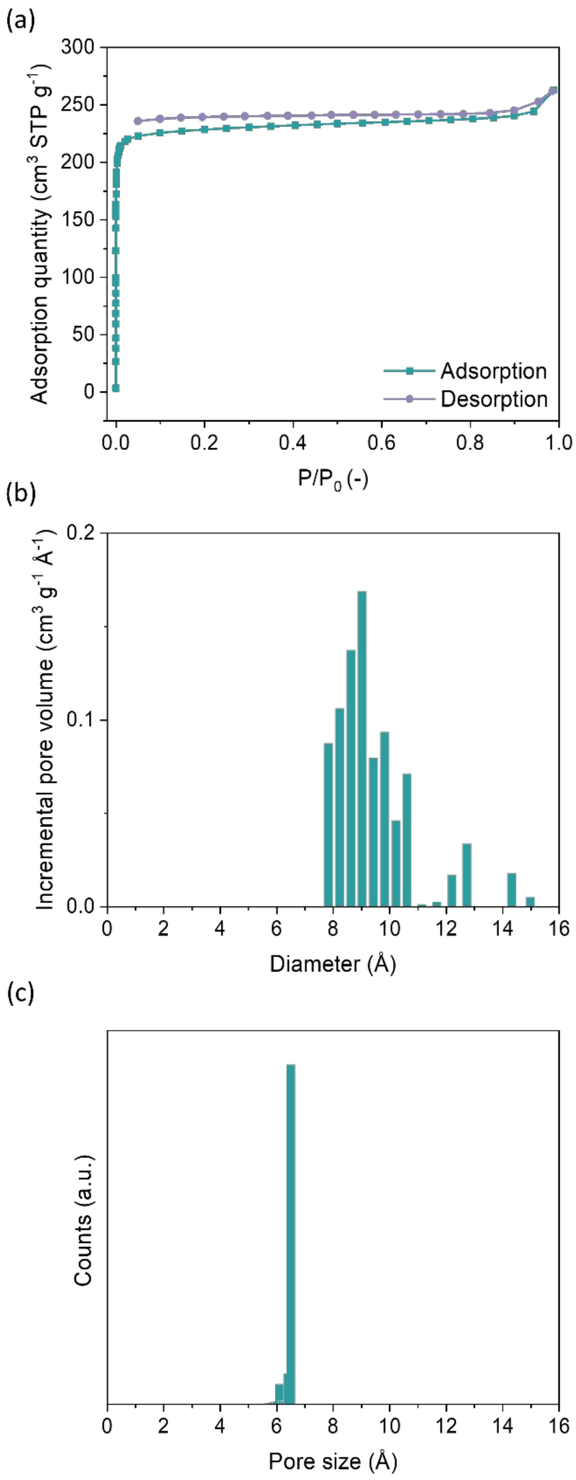

Figure S7. (a) N_2 adsorption-desorption isotherms of MOF-303 at 77 K; (b) pore size distribution of MOF-303 derived from the N_2 adsorption isotherm; (c) simulated pore size distribution of MOF-303 using Zeo++ based on the CIF file CCDC 2078717.

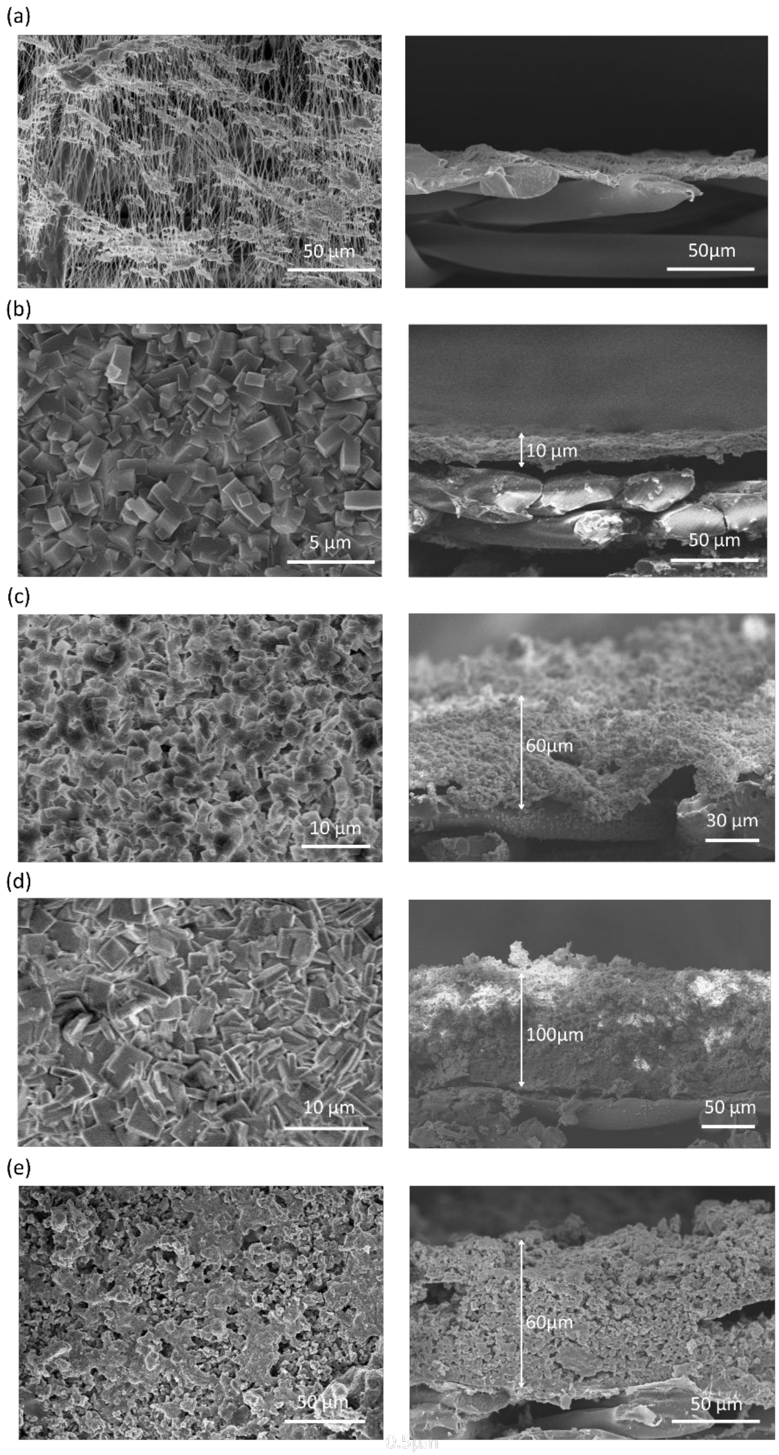

Figure S8. SEM images of (a) bare PVDF substrate, (b-d) MOF-303 membranes with thicknesses of 10, 60, and 100 µm, and (e) MOF-303-CB/PDA-PEDOT-F membrane. The left column shows the top-view images, and the right column shows the side-view images.

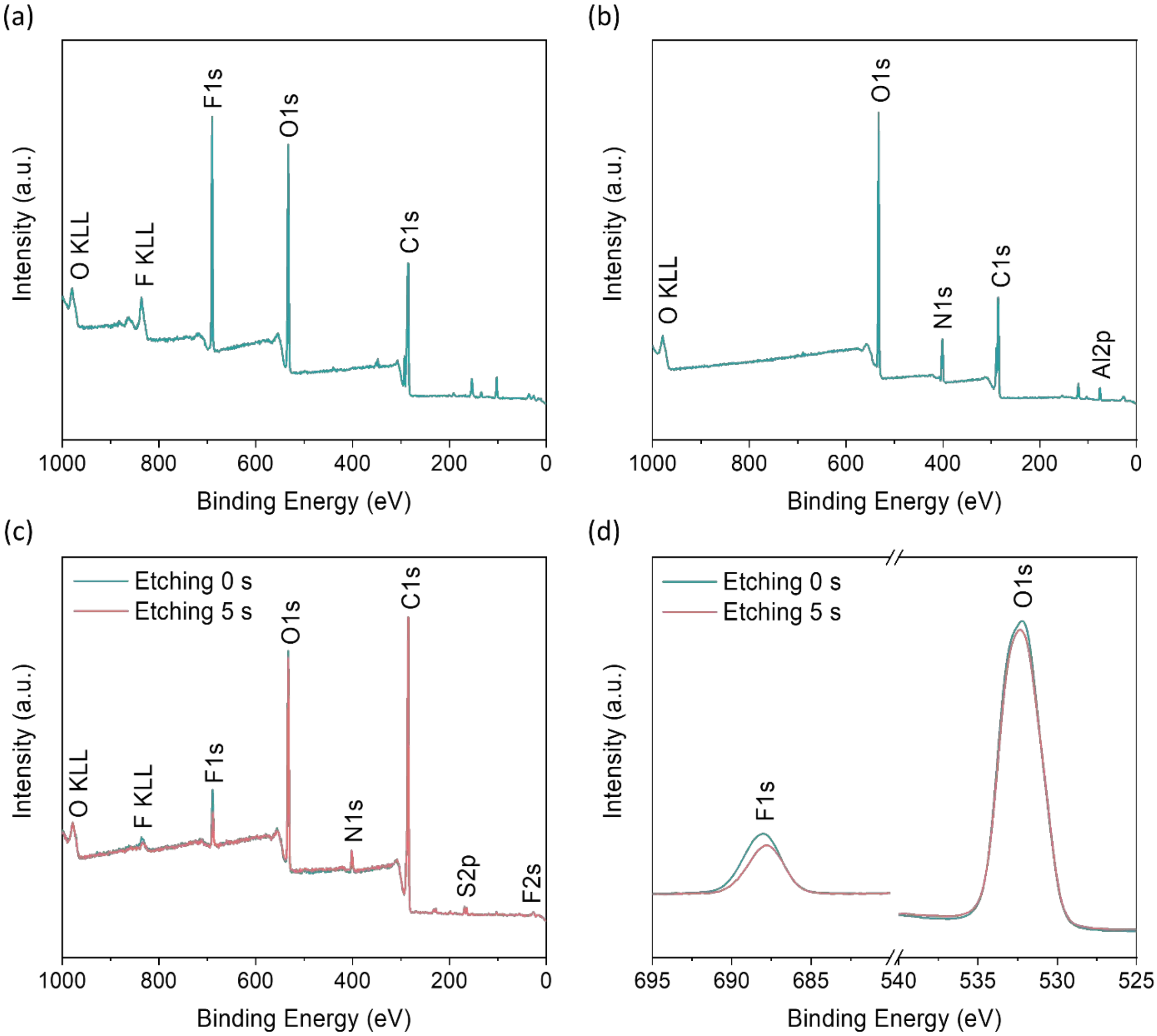

Figure S9. XPS spectra of (a) bare PVDF substrate, (b) MOF-303 membrane on PVDF, and (c) and (d) MOF-303-CB/PDA-PEDOT-F membrane before and after etching for 5 s.

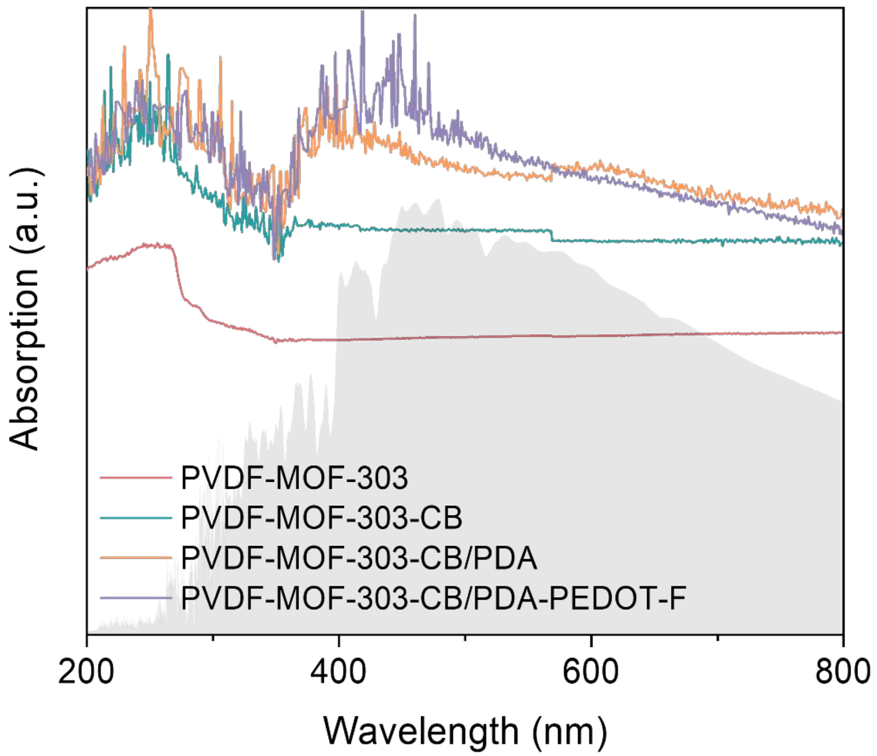

Figure S10. UV-vis spectra of multilayer composition of the MOF-303 membranes. The shaded area indicates the spectrum from solar radiation.

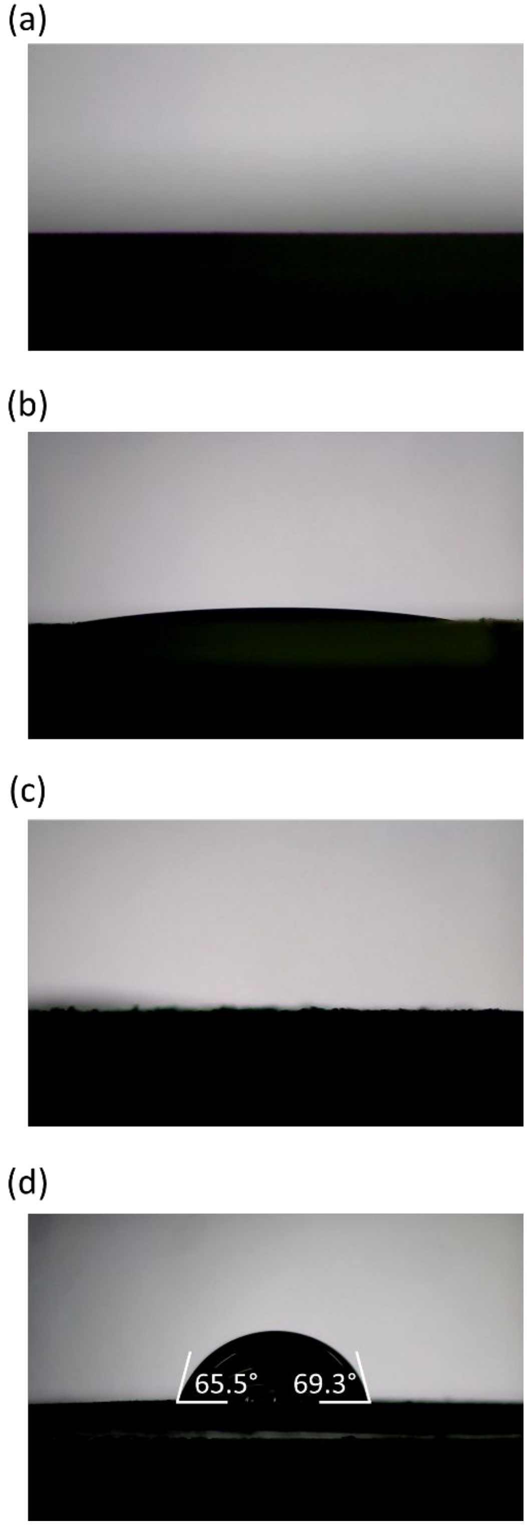

Figure S11. Water contact angle measurements for (a) bare PVDF substrate, (b) MOF-303 on PVDF, (c) MOF-303-CB/PDA membrane, and (d) MOF-303-CB/PDA-PEDOT-F membrane.

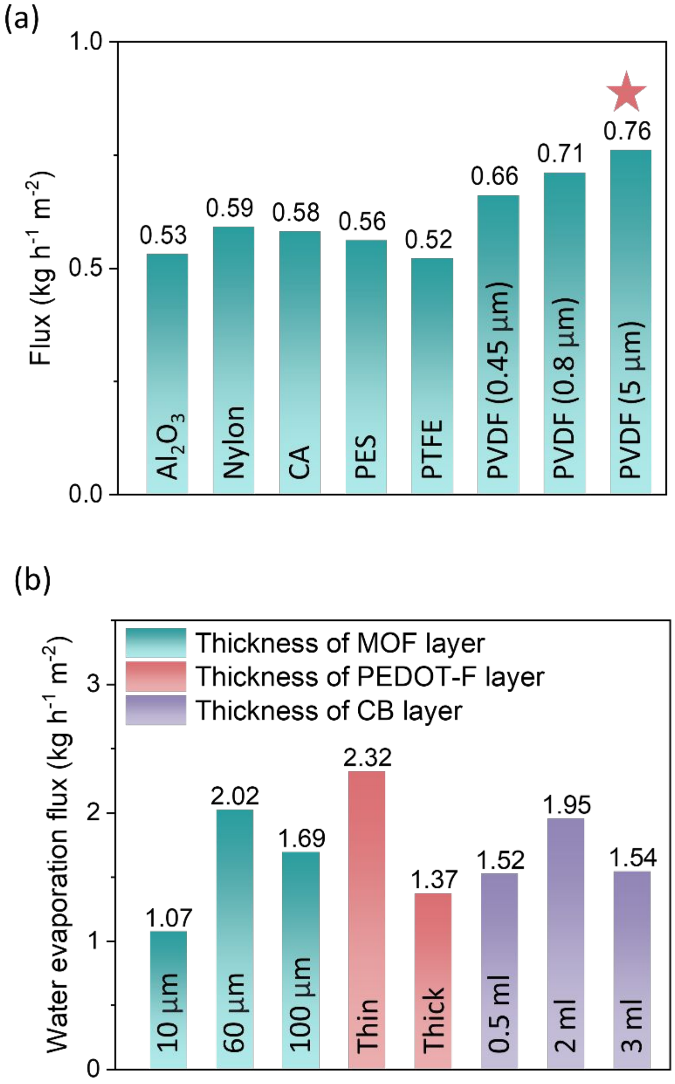

Figure S12. Water evaporation flux from (a) various bare substrates and (b) membranes with different MOF-303, CB/PDA, and PEDOT-F thicknesses. The results for evaluating the effect of MOF-303 thickness were obtained from a device with PVDF (pore size of 5 μm) as the substrate, followed by the deposition of a MOF-303 layer and a CB/PDA layer. The results for evaluating the effect of PEDOT-F thickness were obtained from a device with PVDF (pore size of 5 μm) as the substrate, followed by the deposition of a 60 μm thick MOF-303 layer, a CB/PDA layer, and a PEDOT-F layer.

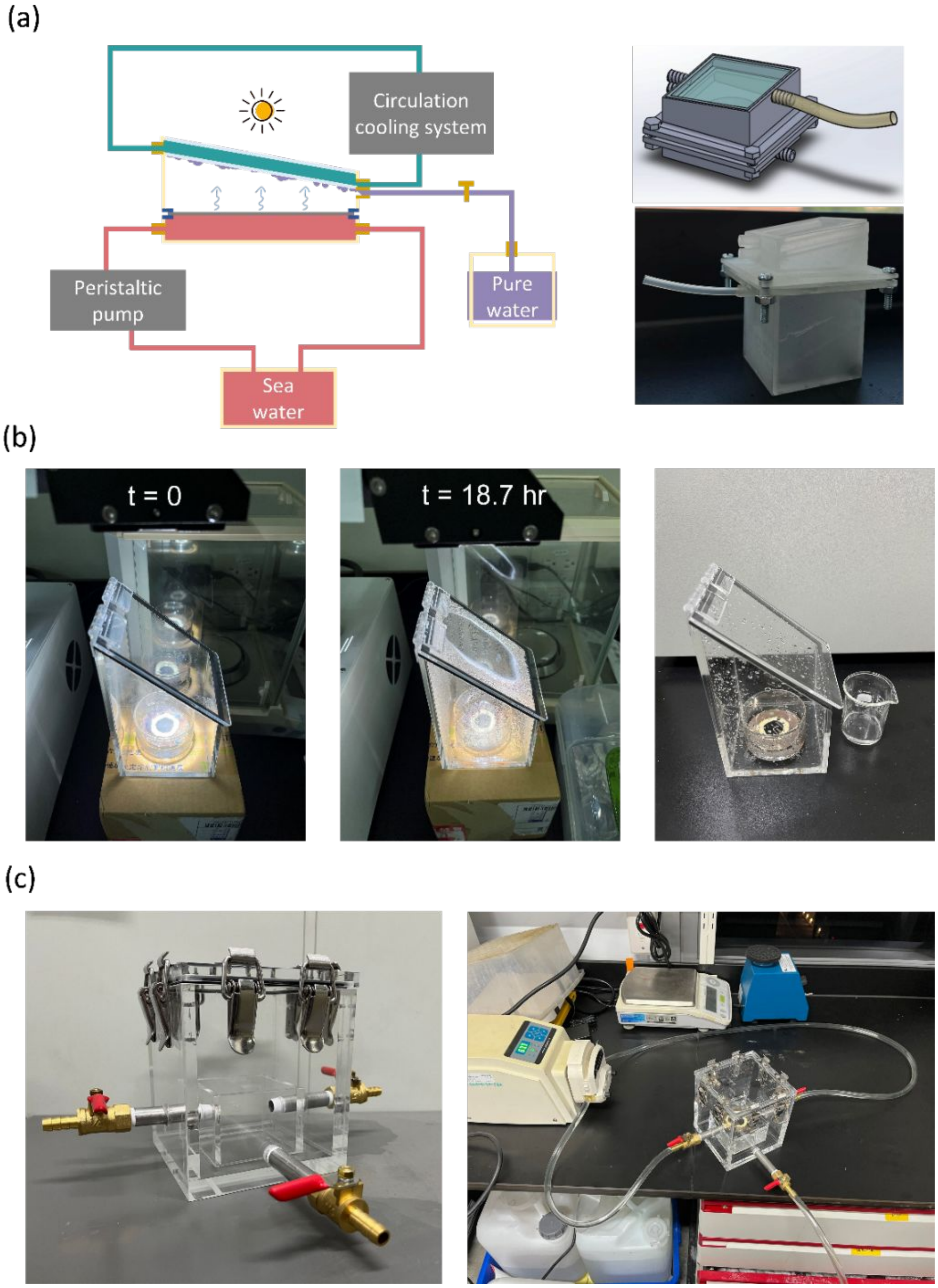

Figure S13. (a) 1st, (b) 2nd, and (c) 3rd versions of the solar steam generation devices proposed in this work.

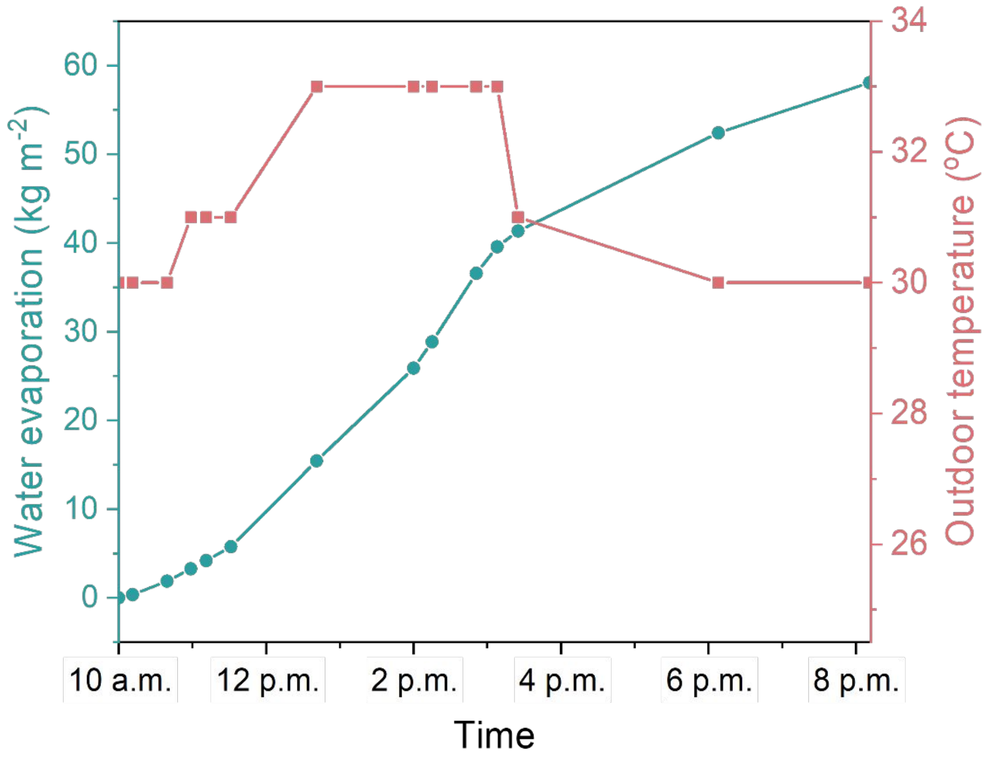

Figure S14. Water evaporation of the optimized MOF-303-CB/PDA-PEDOT-F membrane tested outdoor with the real sunlight.

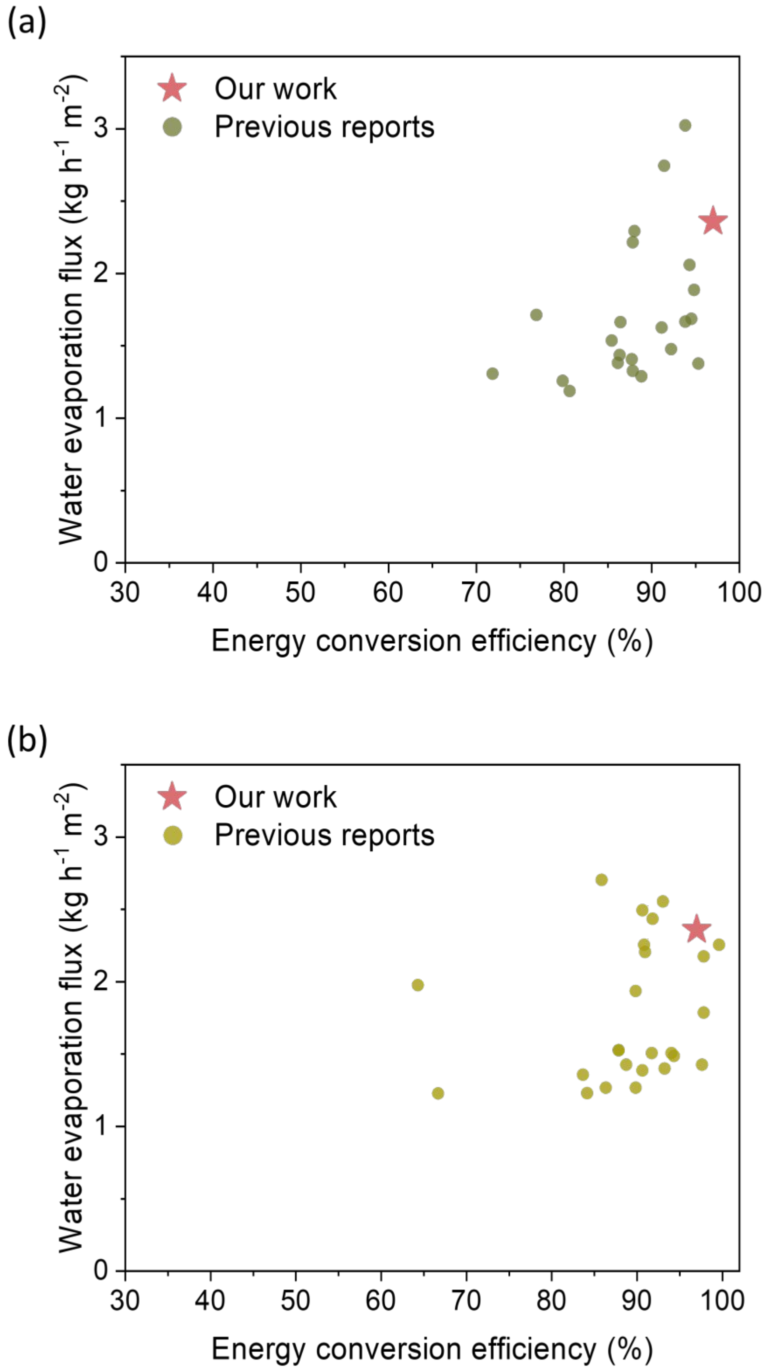

Figure S15. Comparison of device performance for solar steam generation among (a) devices with Janus structures and (b) MOF-based devices. The optimized MOF-303 device reported in this study demonstrates competitive performance.

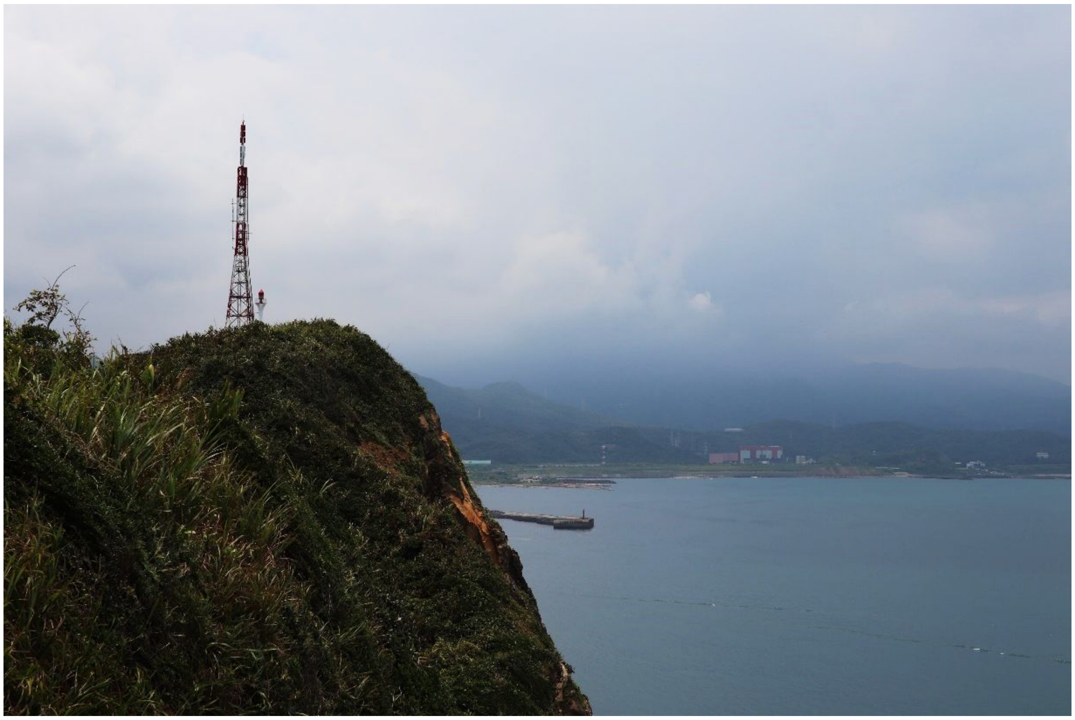

Figure S16. Photograph of the coastline in Keelung, Taiwan, where the seawater for the desalination experiment was obtained.

Supporting Tables

Table S1. Values of the properties for each layer in the multilayer structure used for simulating temperature profiles via the finite element method.

Layer	ρ (kg m^{-3})	C_p (J kg^{-1} K^{-1})	k (W m^{-1} K^{-1})	Porosity
Bulk water	1000	4186	0.6	-
PVDF	284	1510	0.5	0.5
MOF-303	1852	1000	1.5	0.2
CB/PDA	2000	710	0.17	0.5
PEDOT-F	1000	1000	0.15	0.3

Table S2. Flux of solar steam and energy conversion efficiency of previously reported devices under one sun irradiation.

Entry	Materials/Structure	Flux (kg m^{-2} h^{-1})	Efficiency (%)	Reference in SI
1	PVDF-MOF-303-CB/PDA-PEDOT-F	2.32	95	this work
2	N-doped graphene	1.5	80	[1]
3	Thin-film black gold membrane	0.67	47	[2]
4	Ppy coated stainless steel mesh	0.92	58	[3]
5	SH-CGA	1.35	83	[4]
6	Ti_2O_3 nanoparticle on CM	1.32	92.1	[5]
7	Graphene oxide film with 2D water path	1.45	80	[6]
8	Reduced GO-wrapped plant fiber sponges	1.375	88.8	[7]
9	3D graphene network	1.64	91.8	[8]
10	GO-SA-CNT aerogels	1.622	83	[8]
11	HG foams	1.4	91.4	[9]
12	GO leaf	2	83	[10]
13	Carbonized natural woods	12.1	86.7	[11]
14	Black AL-Ti-O hybrid membrane	1.24	77.5	[12]
15	Black amorphous Al-Ti-O nanostructure	1.03	64.46	[12]
16	TiAlON-based nanocomposite	1.13	73	[13]
17	Bilayer wood with a carbonized surface	1	57.3	[14]
18	Carbonized mushrooms	1.475	78	[15]
19	GO/CNT/NFC	1.25	85.6	[16]

20	Flexible wood/CNTs membrane	0.95	65	[17]
21	Narrow-bandgap Ti_2O_3 nanoparticles	1.32	86.5	[18]
22	Graphene oxide-based evaporator	1.27	87.5	[19]
23	Hydrophilic functionalized graphene	0.47	48	[20]
24	Black TiOx nanoparticles	0.8012	50.3	[21]
25	CNT modified filter paper	1.1	73	[22]
26	Polydopamine-filled bacterial nanocellulose	1.13	78	[23]
27	Coke-derived carbons	1.85	62.8	[24]
28	Fe_3O_4@C film	1.03	67	[25]
29	Graphene oxide/SBA-15	1.31	83	[26]
30	MXene Ti_3C_2	1.33	84	[27]
31	Carbon -coated paper	1.28	89	[28]
32	wood roots and rhizomes	1.25	78	[29]
33	rGO/cellous esters membrane	0.84	60	[30]
34	Nylon-CB fabric	1.24	83	[31]
35	CB/PMMA-PAN	1.3	72	[32]
36	CTSe	1.657	86.6	[33]
37	2D Ti_3C_2 MXene membrane	1.31	71	[34]
38	2D and 3D cup-shaped SCS	1.7	96.7	[35]
39	Cu9S5/PVDF membrane	1.173	80.2	[36]
40	CuS/PVDF membrane	1.43	90.4	[37]
41	CuS nanoflowers and semipermeable collodion	1.09	68.6	[38]
42	SWNT/AuNR film	1.6	65	[39]

43	Monolithic polymer foam	1.1687	80.5	[40]
44	Three-dimensional gold nanoflower gel	1.356	85.6	[41]
45	Reduced graphene oxide composite membrane	1.22	80.4	[42]
46	AuNP/PBONF composite film	1.424	83	[43]
47	p-PEGDA-PANi hydrogel	1.4	91.5	[44]
48	Carbon Sponges	1.39	90	[45]
49	Graphite coated wood	1.15	80	[46]
50	CNT -coated tree stump	1	67.8	[47]
51	W18 O49/PDMS mesocrystals membrane	1.15	82	[48]
52	Carbonized melamine foams	1.27	87.3	[49]
53	PVA/Ppy gels	3.2	94	[50]
54	QGF	1.22	84	[51]
55	Porous carbon/paper pulp fiber	1.8	87.6	[52]
56	Carbonized mushroom	1.48	78	[53]
57	TiN/wood-derived carbon foam	1.47	92.5	[54]
58	Carbonized longitudinal wood	1.08	74	[55]
59	Activated carbon fiber cloth	1.59	91.8	[56]
60	Polypyrrole-decorated wood	1.014	72.5	[57]
61	Carbonized balsa wood	0.8	57	[58]
62	Janus evaporator with low tortuosity	1.25	80	[59]
63	Water lily-inspired hierarchical structure	1.31	79.8	[60]
64	HPCM/PHS membrane	1.38	90.8	[61]
65	Polyelectrolyte hydroel foam	1.3	75	[62]

66	CNT/filter paper/cotton thread	1.42	75	[63]
67	Hanging PANi fabric generator	1.94	89.9	[64]
68	EPE foam+air-laid paper	1.46	91.7	[65]
69	EPE foam supported MDPC/SS mesh	1.222	84.3	[66]
70	$K_2Mn_4O_8$ into PVDF	1.18	80.8	[67]
71	rGO/nickel foam	1.38	83.4	[68]
72	Solar thermal photo vapor generator	1.19	90	[69]
73	Graphene-Conjugated Upconversion NPs	0.54	65	[70]
74	PCF	1.52	90	[71]
75	Wood@POF	1.95	80	[72]
76	3D graphene foam	1.3	87	[73]
77	Three dimensional MXene architecture	1.41	88.7	[74]
78	Nitrogen-doped graphene nanopetals	1.21	88.6	[75]
79	Biomimetic MXene textures	1.37	90.1	[76]
80	ALD/Chinese ink with wood	1.31	82.2	[77]
81	Aluminophosphate-treated wood	1.42	90.8	[78]
82	Vertically sliced sugareane internodes	2.5	80	[79]
83	RGO/HNs	1.48	89.2	[80]
84	Corn stalk, MCNTs and TiO_2	2.48	68.2	[81]
85	Conductive ink EL-P-3040+diatomite	1.41	88.6	[82]
86	T-shaped synthetic generator	2.03	75	[83]
87	Biomimetic 3D evaporator	2.63	97	[84]
88	CNF/PLA/PANI hybrid aerogel	1.58	90	[85]

89	pDA-rGO-PTFE	1.45	93.8	[86]
90	Mxene/Co-MOF	1.393	93.4	[87]
91	CTFs/CNT	1.59	93.2	[88]
92	PVDF-HFP/MTTT-BT	1.43	86.5	[89]
93	C/SiO_2/Au aerogel	1.32	80	[90]
94	Black packing polyester (BPP)	0.57	36.7	[91]
95	Loofah porous loofah	1.42	89.9	[92]
96	AlP-compound-treated wood	1.42	90.8	[93]
97	Ti_3C_2-wood	1.465	96	[94]
98	2D Ppy/cotton	1.54	96	[95]
99	PVP and CNTs	1.41	91.1	[96]
100	PPy-AHF	1.8	90	[97]
101	monolithic bilayer membrane	1.07	83.2	[98]
102	Graphite-coated wood	1.15	80	[99]
103	Geopolymer-biomass carbon composite	1.58	84.95	[100]
104	Hollow carbon nanotubes aerogel	1.44	86.8	[101]
105	C@CPVA	1.53	92.38	[102]
106	Ag@PDA wooden flower	2.08	97	[103]
107	Carbonized E. prolifera	1.3	84	[104]
108	Au/disordered nanoporous template	0.8	64	[105]
109	Carbonized tofu	1.65	87.2	[106]
110	Modified black phosphorus nanosheets	0.9437	64.63	[107]
111	SWCNT/gelatin (SGM) membrane	1.39	92	[108]

112	CNFs / Ti_3C_2Tx MXenes	2.287	88.2	[109]
113	Co_3S_4HP/PAN	1.26	86.5	[110]
114	CMPT	1.52	88	[111]
115	F-BP nanosheets	0.9437	64.63	[112]
116	PS/EPE	1.97	64.42	[113]
117	Carbonized corn	1.422	89.3	[114]
118	Candle soot (CS)/adsorbent cotton	1.27	80.7	[115]
119	HN/CNT photothermal paper	1.528	83.2	[116]
120	PSA/PPY cryogel	1.41	96.9	[117]
121	PAN@CuS fabrics	0.61	92.2	[118]
122	MPD	1.7	97.8	[119]
123	PMX	1.41	86.4	[120]
124	cotton-CsxWO3 fabrics	1.58	90.5	[121]
125	KTG	1.47	92.07	[122]
126	TiN NPs	1.01	66.7	[123]
127	PVA and PEDOT:PSS.	2.5	95.5	[124]
128	COFHS DPP-TPA COF/PVA gel	2.5	93.2	[125]
129	GS-COF-1-3d	1.38	95.3	[126]
130	Porous carbon coated wood	2.38	88	[127]
131	Au-rGO coated wood-derived aerogel	1.394	90.1	[128]
132	Bi-layered (BC) bio-foam	1.44	83.5	[129]
133	Ti2O3@PEDOT-OH	2.17	98	[130]
134	TPAD/COF	1.42	94	[131]

135	PVA/GA	1.67	83.4	[132]
136	PANI/ZIF-8 photothermal membrane	1.22	66.8	[133]
137	GT-COF-3-loaded foam	1.314	90.7	[134]
138	Carbonized moldy bread	0.96	71.4	[135]
139	Candle soot (CS)/adsorbent cotton	1.27	80.7	[136]
140	Plasmonic metals	1.51	94.5	[137]
141	COFs	3.02	94	[138]
142	ZIF-L	1.48	94.5	[139]
143	Co-CAT	2.2	91.1	[140]
144	Co-MOF/CNT	2.25	99.8	[141]

Supporting References

(1) Ito, Y.; Tanabe, Y.; Han, J.; Fujita, T.; Tanigaki, K.; Chen, M. Multifunctional Porous Graphene for High-Efficiency Steam Generation by Heat Localization. Advanced Materials 2015, 27 (29), 4302-4307. DOI: 10.1002/adma.201501832.

(2) Bae, K.; Kang, G.; Cho, S. K.; Park, W.; Kim, K.; Padilla, W. J. Flexible thin-film black gold membranes with ultrabroadband plasmonic nanofocusing for efficient solar vapour generation. Nature Communications 2015, 6 (1), 10103. DOI: 10.1038/ncomms10103.

(3) Zhang, L.; Tang, B.; Wu, J.; Li, R.; Wang, P. Hydrophobic Light-to-Heat Conversion Membranes with Self-Healing Ability for Interfacial Solar Heating. Advanced Materials 2015, 27 (33), 4889-4894. DOI: 10.1002/adma.201502362.

(4) Zhu, G.; Xu, J.; Zhao, W.; Huang, F. Constructing Black Titania with Unique Nanocage Structure for Solar Desalination. ACS Applied Materials & Interfaces 2016, 8 (46), 31716-31721. DOI: 10.1021/acsami.6b11466.

(5) Wang, J.; Li, Y.; Deng, L.; Wei, N.; Weng, Y.; Dong, S.; Qi, D.; Qiu, J.; Chen, X.; Wu, T. High-Performance Photothermal Conversion of Narrow-Bandgap Ti₂O₃ Nanoparticles. Advanced Materials 2017, 29 (3), 1603730. DOI: 10.1002/adma.201603730.

(6) Li, X.; Xu, W.; Tang, M.; Zhou, L.; Zhu, B.; Zhu, S.; Zhu, J. Graphene oxide-based efficient and scalable solar desalination under one sun with a confined 2D water path. Proceedings of the National Academy of Sciences 2016, 113 (49), 13953-13958. DOI: 10.1073/pnas.1613031113.

(7) Wang, P.; Zhang, J.; Zeng, Z.; Chen, R.; Huang, X.; Wang, L.; Xu, J.; Hu, Z.; Zhu, Y. Copper iodide as a potential low-cost dopant for spiro-MeOTAD in perovskite solar cells. Journal of Materials Chemistry C 2016, 4 (38), 9003-9008. DOI: 10.1039/c6tc03077g.

(8) Hu, X.; Xu, W.; Zhou, L.; Tan, Y.; Wang, Y.; Zhu, S.; Zhu, J. Tailoring Graphene Oxide-Based Aerogels for Efficient Solar Steam Generation under One Sun. Advanced Materials 2017, 29 (5), 1604031. DOI: 10.1002/adma.201604031.

(9) Ren, H.; Tang, M.; Guan, B.; Wang, K.; Yang, J.; Wang, F.; Wang, M.; Shan, J.; Chen, Z.; Wei, D.; et al. Hierarchical Graphene Foam for Efficient Omnidirectional Solar–Thermal Energy Conversion. Advanced Materials 2017, 29 (38), 1702590. DOI: 10.1002/adma.201702590.

(10) Finnerty, C.; Zhang, L.; Sedlak, D. L.; Nelson, K. L.; Mi, B. Synthetic Graphene Oxide Leaf for Solar Desalination with Zero Liquid Discharge. Environmental Science & Technology 2017, 51 (20), 11701-11709. DOI: 10.1021/acs.est.7b03040.

(11) Jia, C.; Li, Y.; Yang, Z.; Chen, G.; Yao, Y.; Jiang, F.; Kuang, Y.; Pastel, G.; Xie, H.; Yang, B.; et al. Rich Mesostructures Derived from Natural Woods for Solar Steam Generation. Joule 2017, 1 (3), 588-599. DOI: 10.1016/j.joule.2017.09.011.

(12) Yi, L.; Ci, S.; Luo, S.; Shao, P.; Hou, Y.; Wen, Z. Scalable and low-cost synthesis of black amorphous Al-Ti-O nanostructure for high-efficient photothermal desalination. Nano Energy 2017, 41, 600-608. DOI: 10.1016/j.nanoen.2017.09.042.

(13) Liu, H.; Zhang, X.; Hong, Z.; Pu, Z.; Yao, Q.; Shi, J.; Yang, G.; Mi, B.; Yang, B.; Liu, X.; et al. A bioinspired capillary-driven pump for solar vapor generation. Nano Energy 2017, 42, 115-121. DOI: 10.1016/j.nanoen.2017.10.039.

(14) Zhu, M.; Li, Y.; Chen, G.; Jiang, F.; Yang, Z.; Luo, X.; Wang, Y.; Lacey, S. D.; Dai, J.; Wang, C.; et al. Tree-Inspired Design for High-Efficiency Water Extraction. Advanced Materials 2017, 29 (44), 1704107. DOI: 10.1002/adma.201704107.

(15) Xu, N.; Hu, X.; Xu, W.; Li, X.; Zhou, L.; Zhu, S.; Zhu, J. Mushrooms as Efficient Solar Steam-Generation Devices. Advanced Materials 2017, 29 (28), 1606762. DOI: 10.1002/adma.201606762.

(16) Li, Y.; Gao, T.; Yang, Z.; Chen, C.; Luo, W.; Song, J.; Hitz, E.; Jia, C.; Zhou, Y.; Liu, B.; et al. 3D-Printed, All-in-One Evaporator for High-Efficiency Solar Steam Generation under 1 Sun Illumination. Advanced Materials 2017, 29 (26), 1700981. DOI: 10.1002/adma.201700981.

(17) Chen, C.; Li, Y.; Song, J.; Yang, Z.; Kuang, Y.; Hitz, E.; Jia, C.; Gong, A.; Jiang, F.; Zhu, J. Y.; et al. Highly Flexible and Efficient Solar Steam Generation Device. Advanced Materials 2017, 29 (30), 1701756. DOI: 10.1002/adma.201701756.

(18) Zhang, P.; Li, J.; Lv, L.; Zhao, Y.; Qu, L. Vertically Aligned Graphene Sheets Membrane for Highly Efficient Solar Thermal Generation of Clean Water. ACS Nano 2017, 11 (5), 5087-5093. DOI: 10.1021/acsnano.7b01965.

(19) Li, Y.; Gao, T.; Yang, Z.; Chen, C.; Kuang, Y.; Song, J.; Jia, C.; Hitz, E. M.; Yang, B.; Hu, L. Graphene oxide-based evaporator with one-dimensional water transport enabling high-efficiency solar desalination. Nano Energy 2017, 41, 201-209. DOI: 10.1016/j.nanoen.2017.09.034.

(20) Yang, J.; Pang, Y.; Huang, W.; Shaw, S. K.; Schiffbauer, J.; Pillers, M. A.; Mu, X.; Luo, S.; Zhang, T.; Huang, Y.; et al. Functionalized Graphene Enables Highly Efficient Solar Thermal Steam Generation. ACS Nano 2017, 11 (6), 5510-5518. DOI: 10.1021/acsnano.7b00367.

(21) Ye, M.; Jia, J.; Wu, Z.; Qian, C.; Chen, R.; O'Brien, P. G.; Sun, W.; Dong, Y.; Ozin, G. A. Synthesis of Black TiO<i>_x</i> Nanoparticles by Mg Reduction of TiO₂ Nanocrystals and their Application for Solar Water Evaporation. Advanced Energy Materials 2017, 7 (4), 1601811. DOI: 10.1002/aenm.201601811.

(22) Yang, P.; Liu, K.; Chen, Q.; Li, J.; Duan, J.; Xue, G.; Xu, Z.; Xie, W.; Zhou, J. Solar-driven simultaneous steam production and electricity generation from salinity. Energy & Environmental Science 2017, 10 (9), 1923-1927. DOI: 10.1039/c7ee01804e.

(23) Jiang, Q.; Gholami Derami, H.; Ghim, D.; Cao, S.; Jun, Y.-S.; Singamaneni, S. Polydopamine-filled bacterial nanocellulose as a biodegradable interfacial photothermal evaporator for highly efficient solar steam generation. Journal of Materials Chemistry A 2017, 5 (35), 18397-18402. DOI: 10.1039/c7ta04834c.

(24) Wang, J.; Liu, Z.; Dong, X.; Hsiung, C.-E.; Zhu, Y.; Liu, L.; Han, Y. Microporous cokes formed in zeolite catalysts enable efficient solar evaporation. Journal of Materials Chemistry A 2017, 5 (15), 6860-6865. DOI: 10.1039/c7ta00882a.

(25) Chen, R.; Zhu, K.; Gan, Q.; Yu, Y.; Zhang, T.; Liu, X.; Ye, M.; Yin, Y. Interfacial solar heating by self-assembled Fe₃O₄@C film for steam generation. Materials Chemistry Frontiers 2017, 1 (12), 2620-2626. DOI: 10.1039/c7qm00374a.

(26) Shi, L.; Wang, Y.; Zhang, L.; Wang, P. Rational design of a bi-layered reduced graphene oxide film on polystyrene foam for solar-driven interfacial water evaporation. Journal of Materials Chemistry A 2017, 5 (31), 16212-16219. DOI: 10.1039/c6ta09810j.

(27) Li, R.; Zhang, L.; Shi, L.; Wang, P. MXene Ti₃C₂: An Effective 2D Light-to-Heat Conversion Material. ACS Nano 2017, 11 (4), 3752-3759. DOI: 10.1021/acsnano.6b08415.

(28) Liu, Z.; Song, H.; Ji, D.; Li, C.; Cheney, A.; Liu, Y.; Zhang, N.; Zeng, X.; Chen, B.; Gao, J.; et al. Extremely Cost-Effective and Efficient Solar Vapor Generation under Nonconcentrated Illumination Using Thermally Isolated Black Paper. Global Challenges 2017, 1 (2), 1600003. DOI: 10.1002/gch2.201600003.

(29) Liu, H.; Chen, C.; Chen, G.; Kuang, Y.; Zhao, X.; Song, J.; Jia, C.; Xu, X.; Hitz, E.; Xie, H.; et al. High-Performance Solar Steam Device with Layered Channels: Artificial Tree with a Reversed Design. Advanced Energy Materials 2018, 8 (8), 1701616. DOI: 10.1002/aenm.201701616.

(30) Wang, G.; Fu, Y.; Ma, X.; Pi, W.; Liu, D.; Wang, X. Reusable reduced graphene oxide based double-layer system modified by polyethylenimine for solar steam generation. Carbon 2017, 114, 117-124. DOI: 10.1016/j.carbon.2016.11.071.

(31) Jin, Y.; Chang, J.; Shi, Y.; Shi, L.; Hong, S.; Wang, P. A highly flexible and washable nonwoven photothermal cloth for efficient and practical solar steam generation. Journal of Materials Chemistry A 2018, 6 (17), 7942-7949. DOI: 10.1039/c8ta00187a.

(32) Xu, W.; Hu, X.; Zhuang, S.; Wang, Y.; Li, X.; Zhou, L.; Zhu, S.; Zhu, J. Flexible and Salt Resistant Janus Absorbers by Electrospinning for Stable and Efficient Solar Desalination. Advanced Energy Materials 2018, 8 (14), 1702884. DOI: 10.1002/aenm.201702884.

(33) Yang, Y.; Zhao, H.; Yin, Z.; Zhao, J.; Yin, X.; Li, N.; Yin, D.; Li, Y.; Lei, B.; Du, Y.; et al. A general salt-resistant hydrophilic/hydrophobic nanoporous double layer design for efficient and stable solar water evaporation distillation. Materials Horizons 2018, 5 (6), 1143-1150. DOI: 10.1039/c8mh00386f.

(34) Zhao, J.; Yang, Y.; Yang, C.; Tian, Y.; Han, Y.; Liu, J.; Yin, X.; Que, W. A hydrophobic surface enabled salt-blocking 2D Ti₃C₂MXene membrane for efficient and stable solar desalination. Journal of Materials Chemistry A 2018, 6 (33), 16196-16204. DOI: 10.1039/c8ta05569f.

(35) Shi, Y.; Zhang, C.; Li, R.; Zhuo, S.; Jin, Y.; Shi, L.; Hong, S.; Chang, J.; Ong, C.; Wang, P. Solar Evaporator with Controlled Salt Precipitation for Zero Liquid Discharge Desalination. Environmental Science & Technology 2018, 52 (20), 11822-11830. DOI: 10.1021/acs.est.8b03300.

(36) Tao, F.; Zhang, Y.; Yin, K.; Cao, S.; Chang, X.; Lei, Y.; Wang, D.; Fan, R.; Dong, L.; Yin, Y.; et al. A plasmonic interfacial evaporator for high-efficiency solar vapor generation. Sustainable Energy & Fuels 2018, 2 (12), 2762-2769. DOI: 10.1039/c8se00402a.

(37) Tao, F.; Zhang, Y.; Yin, K.; Cao, S.; Chang, X.; Lei, Y.; Wang, D. S.; Fan, R.; Dong, L.; Yin, Y.; et al. Copper Sulfide-Based Plasmonic Photothermal Membrane for High-Efficiency Solar Vapor Generation. ACS Applied Materials & Interfaces 2018, 10 (41), 35154-35163. DOI: 10.1021/acsami.8b11786.

(38) Tao, F.; Zhang, Y.; Cao, S.; Yin, K.; Chang, X.; Lei, Y.; Fan, R.; Dong, L.; Yin, Y.; Chen, X. CuS nanoflowers/semipermeable collodion membrane composite for high-efficiency solar vapor generation. Materials Today Energy 2018, 9, 285-294. DOI: 10.1016/j.mtener.2018.06.003.

(39) Yang, Y.; Yang, X.; Fu, L.; Zou, M.; Cao, A.; Du, Y.; Yuan, Q.; Yan, C.-H. Two-Dimensional Flexible Bilayer Janus Membrane for Advanced Photothermal Water Desalination. ACS Energy Letters 2018, 3 (5), 1165-1171. DOI: 10.1021/acsenergylett.8b00433.

(40) Chen, Q.; Pei, Z.; Xu, Y.; Li, Z.; Yang, Y.; Wei, Y.; Ji, Y. A durable monolithic polymer foam for efficient solar steam generation. Chemical Science 2018, 9 (3), 623-628. DOI: 10.1039/c7sc02967e.

(41) Gao, M.; Peh, C. K.; Phan, H. T.; Zhu, L.; Ho, G. W. Solar Absorber Gel: Localized Macro-Nano Heat Channeling for Efficient Plasmonic Au Nanoflowers Photothermic Vaporization and Triboelectric Generation. Advanced Energy Materials 2018, 8 (25), 1800711. DOI: 10.1002/aenm.201800711.

(42) Wang, Y.; Wang, C.; Song, X.; Megarajan, S. K.; Jiang, H. A facile nanocomposite strategy to fabricate a rGO–MWCNT photothermal layer for efficient water evaporation. Journal of Materials Chemistry A 2018, 6 (3), 963-971. DOI: 10.1039/c7ta08972d.

(43) Chen, M.; Wu, Y.; Song, W.; Mo, Y.; Lin, X.; He, Q.; Guo, B. Plasmonic nanoparticle-embedded poly(<i>p</i>-phenylene benzobisoxazole) nanofibrous composite films for solar steam generation. Nanoscale 2018, 10 (13), 6186-6193. DOI: 10.1039/c8nr01017j.

(44) Yin, X.; Zhang, Y.; Guo, Q.; Cai, X.; Xiao, J.; Ding, Z.; Yang, J. Macroporous Double-Network Hydrogel for High-Efficiency Solar Steam Generation Under 1 sun Illumination. ACS Applied Materials & Interfaces 2018, 10 (13), 10998-11007. DOI: 10.1021/acsami.8b01629.

(45) Zhu, L.; Gao, M.; Peh, C. K. N.; Wang, X.; Ho, G. W. Self-Contained Monolithic Carbon Sponges for Solar-Driven Interfacial Water Evaporation Distillation and Electricity Generation. Advanced Energy Materials 2018, 8 (16), 1702149. DOI: 10.1002/aenm.201702149.

(46) Li, T.; Liu, H.; Zhao, X.; Chen, G.; Dai, J.; Pastel, G.; Jia, C.; Chen, C.; Hitz, E.; Siddhartha, D.; et al. Scalable and Highly Efficient Mesoporous Wood-Based Solar Steam Generation Device: Localized Heat, Rapid Water Transport. Advanced Functional Materials 2018, 28 (16), 1707134. DOI: 10.1002/adfm.201707134.

(47) Wang, Y.; Liu, H.; Chen, C.; Kuang, Y.; Song, J.; Xie, H.; Jia, C.; Kronthal, S.; Xu, X.; He, S.; et al. All Natural, High Efficient Groundwater Extraction via Solar Steam/Vapor Generation. Advanced Sustainable Systems 2019, 3 (1), 1800055. DOI: 10.1002/adsu.201800055.

(48) Chang, Y.; Wang, Z.; Shi, Y.-E.; Ma, X.; Ma, L.; Zhang, Y.; Zhan, J. Hydrophobic W₁₈O₄₉ mesocrystal on hydrophilic PTFE membrane as an efficient solar steam generation device under one sun. Journal of Materials Chemistry A 2018, 6 (23), 10939-10946. DOI: 10.1039/c8ta02700e.

(49) Lin, X.; Chen, J.; Yuan, Z.; Yang, M.; Chen, G.; Yu, D.; Zhang, M.; Hong, W.; Chen, X. Integrative solar absorbers for highly efficient solar steam generation. Journal of Materials Chemistry A 2018, 6 (11), 4642-4648. DOI: 10.1039/c7ta08256h.

(50) Zhao, F.; Zhou, X.; Shi, Y.; Qian, X.; Alexander, M.; Zhao, X.; Mendez, S.; Yang, R.; Qu, L.; Yu, G. Highly efficient solar vapour generation via hierarchically nanostructured gels. Nature Nanotechnology 2018, 13 (6), 489-495. DOI: 10.1038/s41565-018-0097-z.

(51) Shi, Y.; Li, R.; Jin, Y.; Zhuo, S.; Shi, L.; Chang, J.; Hong, S.; Ng, K.-C.; Wang, P. A 3D Photothermal Structure toward Improved Energy Efficiency in Solar Steam Generation. Joule 2018, 2 (6), 1171-1186. DOI: 10.1016/j.joule.2018.03.013.

(52) Kim, K.; Yu, S.; An, C.; Kim, S.-W.; Jang, J.-H. Mesoporous Three-Dimensional Graphene Networks for Highly Efficient Solar Desalination under 1 sun Illumination. ACS Applied Materials & Interfaces 2018, 10 (18), 15602-15608. DOI: 10.1021/acsami.7b19584.

(53) Guo, D.; Yang, X. Highly efficient solar steam generation of low cost TiN/bio-carbon foam. Science China Materials 2019, 62 (5), 711-718. DOI: 10.1007/s40843-018-9353-5.

(54) Liu, P.-F.; Miao, L.; Deng, Z.; Zhou, J.; Su, H.; Sun, L.; Tanemura, S.; Cao, W.; Jiang, F.; Zhao, L.-D. A mimetic transpiration system for record high conversion efficiency in solar steam generator under one-sun. Materials Today Energy 2018, 8, 166-173. DOI: 10.1016/j.mtener.2018.04.004.

(55) Zhang, Y.; Ravi, S. K.; Vaghasiya, J. V.; Tan, S. C. A Barbeque-Analog Route to Carbonize Moldy Bread for Efficient Steam Generation. iScience 2018, 3, 31-39. DOI: 10.1016/j.isci.2018.04.003.

(56) Fang, Q.; Li, T.; Lin, H.; Jiang, R.; Liu, F. Highly Efficient Solar Steam Generation from Activated Carbon Fiber Cloth with Matching Water Supply and Durable Fouling Resistance. ACS Applied Energy Materials 2019, 2 (6), 4354-4361. DOI: 10.1021/acsaem.9b00562.

(57) Wang, Z.; Yan, Y.; Shen, X.; Jin, C.; Sun, Q.; Li, H. A wood–polypyrrole composite as a photothermal conversion device for solar evaporation enhancement. Journal of Materials Chemistry A 2019, 7 (36), 20706-20712. DOI: 10.1039/c9ta04914b.

(58) He, S.; Chen, C.; Kuang, Y.; Mi, R.; Liu, Y.; Pei, Y.; Kong, W.; Gan, W.; Xie, H.; Hitz, E.; et al. Nature-inspired salt resistant bimodal porous solar evaporator for efficient and stable water desalination. Energy & Environmental Science 2019, 12 (5), 1558-1567. DOI: 10.1039/c9ee00945k.

(59) Hu, R.; Zhang, J.; Kuang, Y.; Wang, K.; Cai, X.; Fang, Z.; Huang, W.; Chen, G.; Wang, Z. A Janus evaporator with low tortuosity for long-term solar desalination. Journal of Materials Chemistry A 2019, 7 (25), 15333-15340. DOI: 10.1039/c9ta01576k.

(60) Xu, N.; Li, J.; Wang, Y.; Fang, C.; Li, X.; Wang, Y.; Zhou, L.; Zhu, B.; Wu, Z.; Zhu, S.; et al. A water lily–inspired hierarchical design for stable and efficient solar evaporation of high-salinity brine. Science Advances 2019, 5 (7), eaaw7013. DOI: 10.1126/sciadv.aaw7013.

(61) Ma, X.; Fang, W.; Guo, Y.; Li, Z.; Chen, D.; Ying, W.; Xu, Z.; Gao, C.; Peng, X. Hierarchical Porous SWCNT Stringed Carbon Polyhedrons and PSS Threaded MOF Bilayer Membrane for Efficient Solar Vapor Generation. Small 2019, 15 (15), 1900354. DOI: 10.1002/smll.201900354.

(62) Zeng, J.; Wang, Q.; Shi, Y.; Liu, P.; Chen, R. Osmotic Pumping and Salt Rejection by Polyelectrolyte Hydrogel for Continuous Solar Desalination. Advanced Energy Materials 2019, 9 (38), 1900552. DOI: 10.1002/aenm.201900552.

(63) Xia, Y.; Hou, Q.; Jubaer, H.; Li, Y.; Kang, Y.; Yuan, S.; Liu, H.; Woo, M. W.; Zhang, L.; Gao, L.; et al. Spatially isolating salt crystallisation from water evaporation for continuous solar steam generation and salt harvesting. Energy & Environmental Science 2019, 12 (6), 1840-1847. DOI: 10.1039/c9ee00692c.

(64) Liu, Z.; Wu, B.; Zhu, B.; Chen, Z.; Zhu, M.; Liu, X. Continuously Producing Watersteam and Concentrated Brine from Seawater by Hanging Photothermal Fabrics under Sunlight. Advanced Functional Materials 2019, 29 (43), 1905485. DOI: 10.1002/adfm.201905485.

(65) Gan, Q.; Zhang, T.; Chen, R.; Wang, X.; Ye, M. Simple, Low-Dose, Durable, and Carbon-Nanotube-Based Floating Solar Still for Efficient Desalination and Purification. ACS Sustainable Chemistry & Engineering 2019, 7 (4), 3925-3932. DOI: 10.1021/acssuschemeng.8b05036.

(66) Ma, S.; Qarony, W.; Hossain, M. I.; Yip, C. T.; Tsang, Y. H. Metal-organic framework derived porous carbon of light trapping structures for efficient solar steam generation. Solar Energy Materials and Solar Cells 2019, 196, 36-42. DOI: 10.1016/j.solmat.2019.02.035.

(67) Zhang, Y.; Tao, F.; Cao, S.; Yin, K.; Chang, X.; Fan, R.; Fan, C.; Dong, L.; Yin, Y.; Chen, X. Hierarchical K2Mn4O8 nanoflowers: A novel photothermal conversion material for efficient solar vapor generation. Solar Energy Materials and Solar Cells 2019, 200, 110043. DOI: 10.1016/j.solmat.2019.110043.

(68) Shan, X.; Lin, Y.; Zhao, A.; Di, Y.; Hu, Y.; Guo, Y.; Gan, Z. Porous reduced graphene oxide/nickel foam for highly efficient solar steam generation. Nanotechnology 2019, 30 (42), 425403. DOI: 10.1088/1361-6528/ab3127.

(69) Guo, Y.; Zhou, X.; Zhao, F.; Bae, J.; Rosenberger, B.; Yu, G. Synergistic Energy Nanoconfinement and Water Activation in Hydrogels for Efficient Solar Water Desalination. ACS Nano 2019, 13 (7), 7913-7919. DOI: 10.1021/acsnano.9b02301.

(70) Thakur, M. K.; Gupta, A.; Ghosh, S.; Chattopadhyay, S. Graphene-Conjugated Upconversion Nanoparticles as Fluorescence-Tuned Photothermal Nanoheaters for Desalination. ACS Applied Nano Materials 2019, 2 (4), 2250-2259. DOI: 10.1021/acsanm.9b00186.

(71) Xu, Y.; Liu, D.; Xiang, H.; Ren, S.; Zhu, Z.; Liu, D.; Xu, H.; Cui, F.; Wang, W. Easily scaled-up photo-thermal membrane with structure-dependent auto-cleaning feature for high-efficient solar desalination. Journal of Membrane Science 2019, 586, 222-230. DOI: 10.1016/j.memsci.2019.05.068.

(72) Xia, Z. J.; Yang, H. C.; Chen, Z.; Waldman, R. Z.; Zhao, Y.; Zhang, C.; Patel, S. N.; Darling, S. B. Porphyrin Covalent Organic Framework (POF)-Based Interface Engineering for Solar Steam Generation. Advanced Materials Interfaces 2019, 6 (11), 1900254. DOI: 10.1002/admi.201900254.

(73) Zhang, Q.; Yi, G.; Fu, Z.; Yu, H.; Chen, S.; Quan, X. Vertically Aligned Janus MXene-Based Aerogels for Solar Desalination with High Efficiency and Salt Resistance. ACS Nano 2019, 13 (11), 13196-13207. DOI: 10.1021/acsnano.9b06180.

(74) Zhao, X.; Zha, X.-J.; Pu, J.-H.; Bai, L.; Bao, R.-Y.; Liu, Z.-Y.; Yang, M.-B.; Yang, W. Macroporous three-dimensional MXene architectures for highly efficient solar steam generation. Journal of Materials Chemistry A 2019, 7 (17), 10446-10455. DOI: 10.1039/c9ta00176j.

(75) Wu, S.; Xiong, G.; Yang, H.; Gong, B.; Tian, Y.; Xu, C.; Wang, Y.; Fisher, T.; Yan, J.; Cen, K.; et al. Multifunctional Solar Waterways: Plasma-Enabled Self-Cleaning Nanoarchitectures for Energy-Efficient Desalination. Advanced Energy Materials 2019, 9 (30), 1901286. DOI: 10.1002/aenm.201901286.

(76) Li, K.; Chang, T. H.; Li, Z.; Yang, H.; Fu, F.; Li, T.; Ho, J. S.; Chen, P. Y. Biomimetic MXene Textures with Enhanced Light-to-Heat Conversion for Solar Steam Generation and Wearable Thermal Management. Advanced Energy Materials 2019, 9 (34), 1901687. DOI: 10.1002/aenm.201901687.

(77) Yang, H. C.; Chen, Z.; Xie, Y.; Wang, J.; Elam, J. W.; Li, W.; Darling, S. B. Solar Steam: Chinese Ink: A Powerful Photothermal Material for Solar Steam Generation (Adv. Mater. Interfaces 1/2019). Advanced Materials Interfaces 2019, 6 (1), 1970002. DOI: 10.1002/admi.201970002.

(78) Yang, L.; Chen, G.; Zhang, N.; Xu, Y.; Xu, X. Sustainable Biochar-Based Solar Absorbers for High-Performance Solar-Driven Steam Generation and Water Purification. ACS Sustainable Chemistry & Engineering 2019, 7 (23), 19311-19320. DOI: 10.1021/acssuschemeng.9b06169.

(79) Zhang, W.; Chen, X.; Zhang, G.; Li, J.; Ji, Q.; Hu, C.; Ren, Z. J.; Liu, H.; Qu, J. A salt-rejecting anisotropic structure for efficient solar desalination <i>via</i> heat–mass flux decoupling. Journal of Materials Chemistry A 2020, 8 (24), 12089-12096. DOI: 10.1039/d0ta04326e.

(80) Xiong, Z.-C.; Zhu, Y.-J.; Qin, D.-D.; Yang, R.-L. Flexible Salt-Rejecting Photothermal Paper Based on Reduced Graphene Oxide and Hydroxyapatite Nanowires for High-Efficiency Solar Energy-Driven Vapor Generation and Stable Desalination. ACS Applied Materials & Interfaces 2020, 12 (29), 32556-32565. DOI: 10.1021/acsami.0c05986.

(81) Sun, Z.; Li, W.; Song, W.; Zhang, L.; Wang, Z. A high-efficiency solar desalination evaporator composite of corn stalk, Mcnts and TiO₂: ultra-fast capillary water moisture transportation and porous bio-tissue multi-layer filtration. Journal of Materials Chemistry A 2020, 8 (1), 349-357. DOI: 10.1039/c9ta10898j.

(82) Liu, C.; Cai, C.; Zhao, X. Overcoming Salt Crystallization During Solar Desalination Based on Diatomite-Regulated Water Supply. ACS Sustainable Chemistry & Engineering 2020, 8 (3), 1548-1554. DOI: 10.1021/acssuschemeng.9b06102.

(83) Shao, Y.; Tang, J.; Li, N.; Sun, T.; Yang, L.; Chen, D.; Zhi, H.; Wang, D.; Liu, H.; Xue, G. Designing a bioinspired synthetic tree by unidirectional freezing for simultaneous solar steam generation and salt collection. EcoMat 2020, 2 (1). DOI: 10.1002/eom2.12018.

(84) Wu, L.; Dong, Z.; Cai, Z.; Ganapathy, T.; Fang, N. X.; Li, C.; Yu, C.; Zhang, Y.; Song, Y. Highly efficient three-dimensional solar evaporator for high salinity desalination by localized crystallization. Nature Communications 2020, 11 (1). DOI: 10.1038/s41467-020-14366-1.

(85) Li, S.; He, Y.; Guan, Y.; Liu, X.; Liu, H.; Xie, M.; Zhou, L.; Wei, C.; Yu, C.; Chen, Y. Cellulose Nanofibril-Stabilized Pickering Emulsion and In Situ Polymerization Lead to Hybrid Aerogel for High-Efficiency Solar Steam Generation. ACS Applied Polymer Materials 2020, 2 (11), 4581-4591. DOI: 10.1021/acsapm.0c00674.

(86) Bai, B.; Yang, X.; Tian, R.; Wang, X.; Wang, H. A high efficiency solar steam generation system with using residual heat to enhance steam escape. Desalination 2020, 491, 114382. DOI: 10.1016/j.desal.2020.114382.

(87) Fan, X.; Yang, Y.; Shi, X.; Liu, Y.; Li, H.; Liang, J.; Chen, Y. A MXene-Based Hierarchical Design Enabling Highly Efficient and Stable Solar-Water Desalination with Good Salt Resistance. Advanced Functional Materials 2020, 30 (52), 2007110. DOI: 10.1002/adfm.202007110.

(88) Guo, L.; Gong, J.; Song, C.; Zhao, Y.; Tan, B.; Zhao, Q.; Jin, S. Donor–Acceptor Charge Migration System of Superhydrophilic Covalent Triazine Framework and Carbon Nanotube toward High Performance Solar Thermal Conversion. ACS Energy Letters 2020, 5 (4), 1300-1306. DOI: 10.1021/acsenergylett.0c00394.

(89) Li, H.; Wen, H.; Li, J.; Huang, J.; Wang, D.; Tang, B. Z. Doping AIE Photothermal Molecule into All-Fiber Aerogel with Self-Pumping Water Function for Efficiency Solar Steam Generation. ACS Applied Materials & Interfaces 2020, 12 (23), 26033-26040. DOI: 10.1021/acsami.0c06181.

(90) Zhan, H.-J.; Chen, J.-F.; Zhao, H.-Y.; Jiao, L.; Liu, J.-W.; Yu, S.-H. Biomimetic Difunctional Carbon-Nanotube-Based Aerogels for Efficient Steam Generation. ACS Applied Nano Materials 2020, 3 (5), 4690-4698. DOI: 10.1021/acsanm.0c00683.

(91) Tao, F.; Valenzuela Garcia, A.; Xiao, T.; Zhang, Y.; Yin, Y.; Chen, X. Interfacial Solar Vapor Generation: Introducing Students to Experimental Procedures and Analysis for Efficiently Harvesting Energy and Generating Vapor at the Air–Water Interface. Journal of Chemical Education 2020, 97 (4), 1093-1100. DOI: 10.1021/acs.jchemed.9b00643.

(92) Liu, C.; Hong, K.; Sun, X.; Natan, A.; Luan, P.; Yang, Y.; Zhu, H. An ‘antifouling’ porous loofah sponge with internal microchannels as solar absorbers and water pumpers for thermal desalination. Journal of Materials Chemistry A 2020, 8 (25), 12323-12333. DOI: 10.1039/d0ta03872e.

(93) Chen, T.; Wu, Z.; Liu, Z.; Aladejana, J. T.; Wang, X.; Niu, M.; Wei, Q.; Xie, Y. Hierarchical Porous Aluminophosphate-Treated Wood for High-Efficiency Solar Steam Generation. ACS Applied Materials & Interfaces 2020, 12 (17), 19511-19518. DOI: 10.1021/acsami.0c01815.

(94) Ma, N.; Fu, Q.; Hong, Y.; Hao, X.; Wang, X.; Ju, J.; Sun, J. Processing Natural Wood into an Efficient and Durable Solar Steam Generation Device. ACS Applied Materials & Interfaces 2020, 12 (15), 18165-18173. DOI: 10.1021/acsami.0c02481.

(95) Wang, Z.; Han, M.; He, F.; Peng, S.; Darling, S. B.; Li, Y. Versatile coating with multifunctional performance for solar steam generation. Nano Energy 2020, 74, 104886. DOI: 10.1016/j.nanoen.2020.104886.

(96) Shen, C.; Zhu, Y.; Xiao, X.; Xu, X.; Chen, X.; Xu, G. Economical Salt-Resistant Superhydrophobic Photothermal Membrane for Highly Efficient and Stable Solar Desalination. ACS Applied Materials & Interfaces 2020, 12 (31), 35142-35151. DOI: 10.1021/acsami.0c11332.

(97) Wang, Y.; Liang, W.; Liang, F.; Wang, C.; Song, X.; Huang, M.; Jiang, H. Wettable photothermal hollow fibers arrays for efficient solar-driven desalination under omnidirectional illumination without salt precipitation. Materials Today Energy 2020, 16, 100391. DOI: 10.1016/j.mtener.2020.100391.

(98) Kim, M.; Yang, K.; Kim, Y. S.; Won, J. C.; Kang, P.; Kim, Y. H.; Kim, B. G. Laser-induced photothermal generation of flexible and salt-resistant monolithic bilayer membranes for efficient solar desalination. Carbon 2020, 164, 349-356. DOI: 10.1016/j.carbon.2020.03.059.

(99) Li, W.; Li, X.; Chang, W.; Wu, J.; Liu, P.; Wang, J.; Yao, X.; Yu, Z.-Z. Vertically aligned reduced graphene oxide/Ti3C2Tx MXene hybrid hydrogel for highly efficient solar steam generation. Nano Research 2020, 13 (11), 3048-3056. DOI: 10.1007/s12274-020-2970-y.

(100) Han, S.; Ruoko, T. P.; Gladisch, J.; Erlandsson, J.; Wågberg, L.; Crispin, X.; Fabiano, S. Cellulose-Conducting Polymer Aerogels for Efficient Solar Steam Generation. Advanced Sustainable Systems 2020, 4 (7), 2000004. DOI: 10.1002/adsu.202000004.

(101) Zhao, X.; Zha, X.-J.; Tang, L.-S.; Pu, J.-H.; Ke, K.; Bao, R.-Y.; Liu, Z.-Y.; Yang, M.-B.; Yang, W. Self-assembled core-shell polydopamine@MXene with synergistic solar absorption capability for highly efficient solar-to-vapor generation. Nano Research 2020, 13 (1), 255-264. DOI: 10.1007/s12274-019-2608-0.

(102) Wilson, H. M.; Tushar; Raheman Ar, S.; Jha, N. Plant-derived carbon nanospheres for high efficiency solar-driven steam generation and seawater desalination at low solar intensities. Solar Energy Materials and Solar Cells 2020, 210, 110489. DOI: 10.1016/j.solmat.2020.110489.

(103) Chen, S.; Sun, Z.; Xiang, W.; Shen, C.; Wang, Z.; Jia, X.; Sun, J.; Liu, C.-J. Plasmonic wooden flower for highly efficient solar vapor generation. Nano Energy 2020, 76, 104998. DOI: 10.1016/j.nanoen.2020.104998.

(104) Chen, X.; Meng, C.; Wang, Y.; Zhao, Q.; Li, Y.; Chen, X.-M.; Yang, D.; Li, Y.; Zhou, Y. Laser-Synthesized Rutile TiO₂ with Abundant Oxygen Vacancies for Enhanced Solar Water Evaporation. ACS Sustainable Chemistry & Engineering 2020, 8 (2), 1095-1101. DOI: 10.1021/acssuschemeng.9b05952.

(105) Zhou, X.; Guo, Y.; Zhao, F.; Shi, W.; Yu, G. Topology-Controlled Hydration of Polymer Network in Hydrogels for Solar-Driven Wastewater Treatment. Advanced Materials 2020, 32 (52), 2007012. DOI: 10.1002/adma.202007012.

(106) Liang, J.; Jiang, C.; Wu, W. Printed flexible supercapacitor: Ink formulation, printable electrode materials and applications. Applied Physics Reviews 2021, 8 (2), 021319. DOI: 10.1063/5.0048446.

(107) Sun, B.; Wu, F.; Zhang, Q.; Chu, X.; Wang, Z.; Huang, X.; Li, J.; Yao, C.; Zhou, N.; Shen, J. Insight into the effect of particle size distribution differences on the antibacterial activity of carbon dots. Journal of Colloid and Interface Science 2021, 584, 505-519. DOI: 10.1016/j.jcis.2020.10.015.

(108) Yu, X.; Zhang, Q.; Liu, X.; Xu, N.; Zhou, L. Salt-Resistive Photothermal Materials and Microstructures for Interfacial Solar Desalination. Frontiers in Energy Research 2021, 9. DOI: 10.3389/fenrg.2021.721407.

(109) Han, X.; Ding, S.; Fan, L.; Zhou, Y.; Wang, S. Janus biocomposite aerogels constituted of cellulose nanofibrils and MXenes for application as single-module solar-driven interfacial evaporators. Journal of Materials Chemistry A 2021, 9 (34), 18614-18622. DOI: 10.1039/d1ta04991g.

(110) Yin, X.; Zhang, Y.; Xu, X.; Wang, Y. Bilayer fiber membrane electrospun from MOF derived Co3S4 and PAN for solar steam generation induced sea water desalination. Journal of Solid State Chemistry 2021, 303, 122423. DOI: 10.1016/j.jssc.2021.122423.

(111) Wang, J.; Wang, W.; Feng, L.; Yang, J.; Li, W.; Shi, J.; Lei, T.; Wang, C. A salt-free superhydrophilic metal-organic framework photothermal textile for portable and efficient solar evaporator. Solar Energy Materials and Solar Cells 2021, 231, 111329. DOI: 10.1016/j.solmat.2021.111329.

(112) Li, Z.; Cai, W.; Wang, X.; Hu, Y.; Gui, Z. Self-floating black phosphorous nanosheets as a carry-on solar vapor generator. Journal of Colloid and Interface Science 2021, 582, 496-505. DOI: 10.1016/j.jcis.2020.08.073.

(113) Chen, G.; Jiang, Z.; Li, A.; Chen, X.; Ma, Z.; Song, H. Cu-based MOF-derived porous carbon with highly efficient photothermal conversion performance for solar steam evaporation. Journal of Materials Chemistry A 2021, 9 (31), 16805-16813. DOI: 10.1039/d1ta03695e.

(114) Feng, J.; Bai, B.; Yang, L.; Hu, N.; Wang, H. Low-cost and facile hydrophilic amplification of raw corn straws for the applications of highly efficient interfacial solar steam generation. Materials Chemistry and Physics 2021, 271, 124904. DOI: 10.1016/j.matchemphys.2021.124904.

(115) Zhang, L.; Bai, B.; Hu, N.; Wang, H. Low-cost and facile fabrication of a candle soot/adsorbent cotton 3D-interfacial solar steam generation for effective water evaporation. Solar Energy Materials and Solar Cells 2021, 221, 110876. DOI: 10.1016/j.solmat.2020.110876.

(116) Nawaz, F.; Yang, Y.; Zhao, S.; Sheng, M.; Pan, C.; Que, W. Innovative salt-blocking technologies of photothermal materials in solar-driven interfacial desalination. Journal of Materials Chemistry A 2021, 9 (30), 16233-16254. DOI: 10.1039/d1ta03610f.

(117) Loo, S.-L.; Vásquez, L.; Zahid, M.; Costantino, F.; Athanassiou, A.; Fragouli, D. 3D Photothermal Cryogels for Solar-Driven Desalination. ACS Applied Materials & Interfaces 2021, 13 (26), 30542-30555. DOI: 10.1021/acsami.1c05087.

(118) Liu, Z.; Zhou, Z.; Wu, N.; Zhang, R.; Zhu, B.; Jin, H.; Zhang, Y.; Zhu, M.; Chen, Z. Hierarchical Photothermal Fabrics with Low Evaporation Enthalpy as Heliotropic Evaporators for Efficient, Continuous, Salt-Free Desalination. ACS Nano 2021, 15 (8), 13007-13018. DOI: 10.1021/acsnano.1c01900.

(119) Wang, Y.; Tang, B.; Han, P.; Qi, G.; Gao, D.; Pu, S.; Tao, S. Adjustable photothermal device induced by magnetic field for efficient solar-driven desalination. EcoMat 2021, 3 (5). DOI: 10.1002/eom2.12139.

(120) Zhang, B.; Gu, Q.; Wang, C.; Gao, Q.; Guo, J.; Wong, P. W.; Liu, C. T.; An, A. K. Self-Assembled Hydrophobic/Hydrophilic Porphyrin-Ti₃C₂T<i>_x</i> MXene Janus Membrane for Dual-Functional Enabled Photothermal Desalination. ACS Applied Materials & Interfaces 2021, 13 (3), 3762-3770. DOI: 10.1021/acsami.0c16054.

(121) Liu, Z.; Zhong, Q.; Wu, N.; Zhou, H.; Wang, L.; Zhu, L.; Jiang, N.; Zhu, B.; Chen, Z.; Zhu, M. Vertically symmetrical evaporator based on photothermal fabrics for efficient continuous desalination through inversion strategy. Desalination 2021, 509, 115072. DOI: 10.1016/j.desal.2021.115072.

(122) Zhang, C.-R.; Cui, W.-R.; Niu, C.-P.; Yi, S.-M.; Liang, R.-P.; Qi, J.-X.; Chen, X.-J.; Jiang, W.; Zhang, L.; Qiu, J.-D. rGO-based covalent organic framework hydrogel for synergistically enhance uranium capture capacity through photothermal desalination. Chemical Engineering Journal 2022, 428, 131178. DOI: 10.1016/j.cej.2021.131178.

(123) Farid, M. U.; Kharraz, J. A.; An, A. K. Plasmonic Titanium Nitride Nano-enabled Membranes with High Structural Stability for Efficient Photothermal Desalination. ACS Applied Materials & Interfaces 2021, 13 (3), 3805-3815. DOI: 10.1021/acsami.0c17154.

(124) Li, C.; Zhu, B.; Liu, Z.; Zhao, J.; Meng, R.; Zhang, L.; Chen, Z. Polyelectrolyte-based photothermal hydrogel with low evaporation enthalpy for solar-driven salt-tolerant desalination. Chemical Engineering Journal 2022, 431, 134224. DOI: 10.1016/j.cej.2021.134224.

(125) Huang, Z.; Luo, Y. H.; Geng, W. Y.; Wan, Y.; Li, S.; Lee, C. S. Marriage of 2D Covalent–Organic Framework and 3D Network as Stable Solar-Thermal Still for Efficient Solar Steam Generation. Small Methods 2021, 5 (5), 2100036. DOI: 10.1002/smtd.202100036.

(126) Chen, Z.; Su, Y.; Tang, X.; Zhang, X.; Duan, C.; Huang, F.; Li, Y. Manipulating Grain Boundary Defects in π-Conjugated Covalent Organic Frameworks Enabling Intrinsic Radical Generation for Photothermal Conversion. Solar RRL 2021, 5 (12), 2100762. DOI: 10.1002/solr.202100762.

(127) Liu, N.; Hao, L.; Zhang, B.; Niu, R.; Gong, J.; Tang, T. Rational Design of High-Performance Bilayer Solar Evaporator by Using Waste Polyester-Derived Porous Carbon-Coated Wood. ENERGY & ENVIRONMENTAL MATERIALS 2022, 5 (2), 617-626. DOI: 10.1002/eem2.12199.

(128) Garemark, J.; Perea-Buceta, J. E.; Rico Del Cerro, D.; Hall, S.; Berke, B.; Kilpeläinen, I.; Berglund, L. A.; Li, Y. Nanostructurally Controllable Strong Wood Aerogel toward Efficient Thermal Insulation. ACS Applied Materials & Interfaces 2022, 14 (21), 24697-24707. DOI: 10.1021/acsami.2c04584.

(129) Wei, Z.; Irshad, M. S.; Arshad, N.; Noureen, L.; Ahmed, I.; Mushtaq, N.; Asghar, M. S.; Hayat, Q.; Ghazanfar, U.; Idrees, M.; et al. Nanocomposite-Enhanced Efficient Evaporation System for Solar-Driven Seawater Desalination—An Optimized Design for Clean Water Production. Nanomaterials 2022, 12 (19), 3296. DOI: 10.3390/nano12193296.

(130) Hu, T.-N.; Hsu, C.-H.; Chiou, D.-S.; Kang, D.-Y.; Luo, S.-C. CAU-10-H as efficient water sorbent for solar steam generation. Journal of the Taiwan Institute of Chemical Engineers 2022, 141, 104593. DOI: 10.1016/j.jtice.2022.104593.

(131) Yan, X.; Lyu, S.; Xu, X. Q.; Chen, W.; Shang, P.; Yang, Z.; Zhang, G.; Chen, W.; Wang, Y.; Chen, L. Superhydrophilic 2D Covalent Organic Frameworks as Broadband Absorbers for Efficient Solar Steam Generation. Angewandte Chemie International Edition 2022, 61 (19). DOI: 10.1002/anie.202201900.

(132) Wang, C.; Wang, Y.; Guan, W.; Wang, P.; Feng, J.; Song, N.; Dong, H.; Yu, L.; Sui, L.; Gan, Z.; et al. A self-floating and integrated bionic mushroom for highly efficient solar steam generation. Journal of Colloid and Interface Science 2022, 612, 88-96. DOI: 10.1016/j.jcis.2021.12.064.

(133) Peng, Y.; Wei, X.; Wang, Y.; Li, W.; Zhang, S.; Jin, J. Metal–Organic Framework Composite Photothermal Membrane for Removal of High-Concentration Volatile Organic Compounds from Water via Molecular Sieving. ACS Nano 2022, 16 (5), 8329-8337. DOI: 10.1021/acsnano.2c02520.

(134) Tang, X.; Chen, Z.; Xu, Q.; Su, Y.; Xu, H.; Horike, S.; Zhang, H.; Li, Y.; Gu, C. Design of Photothermal Covalent Organic Frameworks by Radical Immobilization. CCS Chemistry 2022, 4 (8), 2842-2853. DOI: 10.31635/ccschem.021.202101198.

(135) Wei, Z.; Wang, J.; Guo, S.; Tan, S. C. Towards highly salt-rejecting solar interfacial evaporation: Photothermal materials selection, structural designs, and energy management. Nano Research Energy 2022, 1, e9120014. DOI: 10.26599/nre.2022.9120014.

(136) Bai, Z.; Xu, H.; Yang, B.; Yao, J.; Li, G.; Guo, K.; Wang, N.; Liang, N. Fe3O4/Diatomite-Decorated Cotton Evaporator for Continuous Solar Steam Generation and Water Treatment. Materials 2022, 15 (17), 6110. DOI: 10.3390/ma15176110.

(137) Zhang, Y.; Wang, Y.; Yu, B.; Yin, K.; Zhang, Z. Hierarchically structured black gold film with ultrahigh porosity for solar steam generation. Advanced Materials 2022, 34 (21), 2200108.

(138) Jia, S.; Hao, L.; Liu, Y.; Lin, E.; Liu, W.; Yang, Y.; Tian, Y.; Peng, Y.; Cheng, P.; Chen, Y.; et al. Freestanding Hydrophilic/Hydrophobic Janus Covalent Organic Framework Membranes for Highly Efficient Solar Steam Generation. ACS Materials Letters 2023, 5 (2), 458-465. DOI: 10.1021/acsmaterialslett.2c01056.

(139) Cheng, L.; Zuo, L.; Yan, S.; Shen, J.; Li, C.; Che, Y.; Wang, L.; Anton, R.; Bian, T. In situ growth of polydopamine modified ZIF-L arrays on air-laid paper as flexible evaporator for efficient solar desalination. Desalination 2023, 565, 116832. DOI: 10.1016/j.desal.2023.116832.

(140) He, P.; Lan, H.; Bai, H.; Zhu, Y.; Fan, Z.; Liu, J.; Liu, L.; Niu, R.; Dong, Z.; Gong, J. Rational construction of “all-in-one” metal-organic framework for integrated solar steam generation and advanced oxidation process. Applied Catalysis B: Environmental 2023, 337, 123001. DOI: 10.1016/j.apcatb.2023.123001.

(141) Bai, H.; He, P.; Hao, L.; Fan, Z.; Niu, R.; Tang, T.; Gong, J. Waste-treating-waste: Upcycling discarded polyester into metal–organic framework nanorod for synergistic interfacial solar evaporation and sulfate-based advanced oxidation process. Chemical Engineering Journal 2023, 456, 140994. DOI: 10.1016/j.cej.2022.140994.
